# The perceptual primacy of feeling: Affectless visual machines explain a majority of variance in human visually evoked affect

**DOI:** 10.1073/pnas.2306025121

**Published:** 2025-01-23

**Authors:** Colin Conwell, Daniel Graham, Chelsea Boccagno, Edward A. Vessel

**Affiliations:** ^a^Department of Psychology, Harvard University, Cambridge, MA 02139; ^b^Department of Psychological Science, Hobart and William Smith Colleges; ^c^Department of Psychiatry, Massachusetts General Hospital, Boston, MA 02114; ^d^Department of Epidemiology, Harvard T.H. Chan School of Public Health; ^e^Department of Psychology, City College, City University of New York, New York, NY 10031

**Keywords:** affective science, cognitive neuroscience, visually evoked affect, aesthetics, machine vision

## Abstract

Looking at the world often involves not just seeing things, but feeling things. Modern feedforward machine vision systems that learn to perceive the world in the absence of active physiology, deliberative thought, or any form of feedback that resembles human affective experience offer tools to demystify the relationship between seeing and feeling, and to assess how much of visually evoked affective experiences may be a straightforward function of representation learning over natural image statistics. In this work, we deploy a diverse sample of 180 state-of-the-art deep neural network models trained only on canonical computer vision tasks to predict human ratings of arousal, valence, and beauty for images from multiple categories (objects, faces, landscapes, art) across two datasets. Importantly, we use the features of these models without additional learning, linearly decoding human affective responses from network activity in much the same way neuroscientists decode information from neural recordings. Aggregate analysis across our survey, demonstrates that predictions from purely perceptual models explain a majority of the explainable variance in average ratings of arousal, valence, and beauty alike. Finer-grained analysis within our survey (e.g. comparisons between shallower and deeper layers, or between randomly initialized, category-supervised, and self-supervised models) point to rich, preconceptual abstraction (learned from diversity of visual experience) as a key driver of these predictions. Taken together, these results provide further computational evidence for an information-processing account of visually evoked affect linked directly to efficient representation learning over natural image statistics, and hint at a computational locus of affective and aesthetic valuation immediately proximate to perception.

## Significance

Human visual experience is defined not only by the light reflecting on our eyes (sensation), but by the feelings (affect) we feel concurrently. Psychological theories about where these feelings come from often focus mostly on the role of changes in our bodily states (physiology) or on our conscious thoughts about the things we are seeing (cognition). Far less frequently do these theories focus on the role of seeing itself (perception). In this research, we show that machine vision systems—which have neither bodily states nor conscious thoughts—can predict with remarkable accuracy how humans will feel about the things they look at. This suggests that perceptual processes (built on rich sensory experiences) may shape what we feel about the world around us far more than many psychological theories suggest.

For sentient biological agents, looking at the world almost always means feeling the world. Though often studied in isolation, perception and affect are linked intimately in everyday phenomenological experience (1), at both conscious and subconscious levels (2, 3). Our exposure to a beautiful, moving, inviting, or aversive stimulus perhaps self-evidently evokes processes beyond what the contemporary psychological community typically refers to as vision, but where exactly “seeing” stops and “feeling” begins is a question the holistic nature of human experience makes difficult to answer with brain or behavioral experiments alone.

Here, we attempt to bypass this barrier by using a large survey of visual machines—which only see and cannot feel—to predict human affective responses to a diverse set of natural images. In doing so, our ultimate goal is to better isolate the unique contributions of (visual) perception to (visually evoked) affect. And because definitions are critical to the logic of meeting this goal, we begin with this initial glossary: (visual) perception, which we define as processes that map (visual) sensation (i.e. light reflected on the retina) into mental representations whose primary referents are external objects (e.g. physical matter); affect, which we define both as “core affect” (i.e. arousal and valence, exclusively) and affect more broadly construed [i.e. “anything emotional” (4)]; visually evoked affect, which we define as affect that arises in response to an external (visual) stimulus.

While other definitions abound, many theoretical frameworks of (visually evoked) affect would typically describe “seeing with feeling” (c.f. 2) as the product of 3 interactive processes: perception of the immediate sensory environment (e.g. seeing a bear in a forest), physiological or bodily state change (e.g. increased heartbeat), and cognitive interpretation of the physiological or bodily state change with respect to one’s percepts (e.g. interpreting one’s increased heartbeat to mean “fear of the bear”) (4–6). That some combination of perception, physiology, and cognition undergirds our feelings is a given, but the relative contributions of each to any particular instance of affect remains a matter of deep debate (7–9). Even well-controlled studies that scrutinize the behavioral outputs of affect (and the intermediate brain states that produce them) suffer from the practical impossibility of fully isolating one type of process from another, especially when the subject of study is human.

Visual machines, on the other hand, give us the empirical control to do precisely this: that is, to disentangle perceptual computations from the influence of physiology and cognitive manipulation and isolate those computations we can veridically consider “void of affect.” Again, key to understanding this control is a proper delineation of what these machines are, and what they are not. What these machines generally tend to be are highly specialized, feedforward representation learners designed to transform digital inputs (e.g. the pixels of an image) into one of multiple numerical vectors (e.g. the one-hot embedding of a category label). What these models by definition are not, however, is affective—with no states that correspond under any definition to those of a physiological body, nor the capacity for the abstract symbol manipulations we typically associate with deliberative thought or cognition.

The power of these machines as empirical tools for studying “seeing with feeling,” then, is in their ability to “see” without “feeling” anything at all. Once trained, their outputs serve effectively as a coordinate system for triangulating the location of a stimulus in a representational space circumscribed entirely by four constraints: input, architecture, task, and learning rule. By using these coordinates to predict what humans will feel in response to visual stimuli, we can begin to approximate not only how much of visually evoked affect may be supported by perception alone but also how purely perceptual processes could support affective experience in the first place.

Candidate answers to these questions have long been proposed in the literature of several fields. Decades before the building of a “seeing” machine became a tangible reality, the “affective computing” movement (10, 11) made great strides in conceptualizing the ways an (artificially) intelligent system might experience affect as a function of perceptual sense-making. Computational aesthetics research has been another significant contributor to the idea [hearkening back even to Kant’s “universal subjective” (12)] that what we feel may be modeled directly as a function of what we see: In recent years, for example, such research has shown that image-computable statistics and features extracted from visual machines can successfully distinguish art from nonart (13–18) and are directly able to predict a certain degree of aesthetic liking and beauty (19–28). In affective science more broadly, we see models such as “Emo-Net” (29), a modified machine vision system that uses similar image-computable feature extraction pipelines, predicting a large variety of other affective and emotional responses (e.g. fear, surprise).

Other precedents may be found in applied machine learning, where the simultaneous advent of broadly available computing power and large-scale datasets of human image ratings [e.g. the AVA dataset (30)] has now allowed for the engineering of models trained directly to predict human affective and aesthetic responses to images (31–40). These models have also been employed to optimize graphic design or aid with visual composition (41, 42)—a trend that has become particularly relevant with the recent resurgence of algorithmic text-to-image pipelines and other “generative AI” systems (e.g. OpenAI’s Dall.E3 and MidJourney) (43, 44).

These many diverse research efforts have given us solid methodological groundwork for exploring the intersection of perceptual computation and affect, but none in and of themselves answer the questions of just how far we can go in predicting affect with perceptual computations alone, and why. Our work attempts to fill this gap by employing a comprehensive survey of 180 diverse, state-of-the-art machine vision systems to determine the statistical upper limit on affective prediction from perceptual computations alone, and use the variation across these models (i.e. in terms of architecture, task, and input) to develop a deeper understanding of how such prediction is possible (45–47). This “model zoology” approach—applied similarly in recent neuroscientific works (48, 49)—allows us to unify the seminal groundwork of affective (neuro)science, computational aesthetics, and machine learning in a single unified pipeline, and affords us deep statistical confidence in three key claims:

- The purely perceptual computations of “affect-less” visual machines are sufficient for explaining the majority of explainable variance in human affective responses to images.
- These affectless machines predict the majority of explainable variance not only in arousal and valence ratings (often considered more “physiological” in nature) but also in beauty ratings [often considered more “cognitive” in nature (50)].
- The ability of these machines to predict these responses is not solely a function of their ability to efficiently encode the statistical properties of individual images but is instead a function of the representations they learn from experience over many images—in other words, their hierarchically structured knowledge of the visual world.

Taken together, our results suggest that perceptual learning over natural image statistics may occupy a more central place in the ontology of visually evoked affect than previous theories have suggested.

## 1. Results

**Fig. 1.**
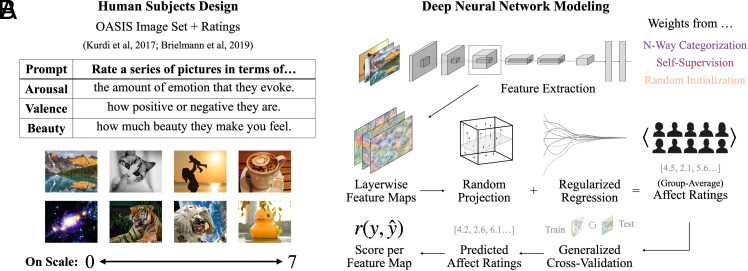
Overview of our approach. (*A*) shows a schematic of our the target human behavioral data. Respondents in the human behavioral paradigm were asked (via one of 3 prompts, corresponding to the 3 affect categories) to provide ratings for a series of images on a scale of 0 to 7. The average of these ratings (per image) is the target of our decoding. (*B*) shows a schematic of the decoding pipeline. To decode the group-average affect for a given image, we extract the features for that image at each distinct computational submodule (layer) of a candidate deep neural network model, producing a series of layerwise feature maps, the dimensionality of which we reduce with sparse random projection. We then use these dimensionality-reduced features as the predictors in a leave-one-out cross-validated, regularized regression wherein the output is a prediction of the group-average affect rating per image. We then correlate these predictions with the actual ratings across images to obtain a single accuracy value per feature map (model layer).

Our general approach (Fig. 1) is to fit linear decoding models to the features extracted from every layer of 180 distinct deep neural network models to assess the extent to which affect-predictive information is inherent to these features, despite these features never having been shaped to predict affective experiences per se. We do this for two datasets: the open affective standardized image set (OASIS) images [900 images with ratings of arousal, valence, and beauty for each; (51, 52)] and the Vessel dataset [562 images with ratings of beauty only; (53)]. We contextualize the predictive accuracies of these linear decoders by comparing their performance to two measures of interrater variability: the mean-minus-one correlation (i.e. the correlation of each respondent’s ratings to the group-average ratings minus that respondent, henceforth $r_{\text{MM1}}$) and the Spearman–Brown split-half reliability (henceforth $r_{\text{split}}$). $r_{\text{MM1}}$ we interpret (with some caveats) as a “ceiling of shared taste” (c.f. refs. 53 and 54); $r_{\text{split}}$ we interpret as a more traditional “noise ceiling.” Both of these thresholds allow us to quantify how accurate our models’ predictions are, given how accurate they could in practice be based on the level of agreement between human respondents. Intuitively, if all human respondents agree, both of these ceilings would be 1; if all human respondents disagree, this ceiling would be 0. (Details on all aspects of our approach may be found in *Materials and Methods*; details on the collection of the behavioral ratings data may be found in *SI Appendix*, section 1).

To “score” how accurately we can predict affect from a candidate feature space, we use two metrics: The first is the Pearson correlation between the predicted ratings decoded from the model, and the actual affect ratings provided by the human respondents (henceforth ($r_{\left(y,\hat{y}\right)}$). The second, which we refer to as “explainable variance explained” (henceforth $r_{\text{EVE}}^{2}$) is the squared Pearson correlation between predicted and actual ratings, divided by the squared Spearman–Brown split-half reliability:

[1] $$r_{\text{EVE}}^{2}=\frac{r_{\left(y,\hat{y}\right)}^{2}}{r_{\text{split}}^{2}}$$

This second metric, a variant of the “noise-normalized” or “noise-corrected” scores commonly used in the “neural benchmarking” literature (e.g., refs. 55–57; see also ref. 58) converts our first-order accuracy measure ($r_{\left(y,\hat{y}\right)}$) into a proportion of the variance in the data that is not otherwise attributable to noise (or in this case, to human disagreement not otherwise attributable to the stimuli). (For more discussion of this choice of metrics, as well as results provided in a more standard “explained variance score,” see *SI Appendix*, section 2).

Unless otherwise noted, we use the following convention in the reporting of means: arithmetic mean [lower 95% bootstrapped CI, upper 95% bootstrapped CI]. A glossary with the expanded forms of metric notation, dataset, and model acronyms may be found in *SI Appendix*, section 1.

### 1.1. How Accurately Can We Decode Affect from Purely Perceptual Models?

Here, we address the central question of this work, reporting both the average and highest affect-predictive accuracies of the (ImageNet-trained) object recognition networks, which in this analysis we treat as the canonical case of a purely perceptual model.^*^

#### 1.1.1. The average perceptual model predicts group-level affect far above the ceiling of “shared taste,” and over halfway to the overall noise ceiling.

Object recognition models perform well in the prediction of group-level affect ratings across all 3 affect categories (arousal, valence, and beauty) and across both datasets surveyed. For the OASIS dataset, mean predictive accuracies ($r_{\left(y,\hat{y}\right)}$) across the N = 72 object recognition models are 0.688 [0.679, 0.697] for arousal; 0.663 [0.652, 0.675] for valence; and 0.746 [0.736, 0.755] for beauty. For the Vessel dataset (beauty alone), mean predictive accuracy is 0.671 [0.665, 0.678].

To better contextualize these accuracies, we consider two forms of variability in the human respondent ratings: the mean-minus-one correlations ($r_{\text{MM1}}$) and the Spearman–Brown split-half reliability ($r_{\text{split}}$). The first of these, $r_{\text{MM1}}$, gives us a sense of the overall agreement among human respondents for a given set of affect ratings. In the OASIS dataset, the mean $r_{\text{MM1}}$ across respondents is 0.481 [0.458, 0.504] for arousal, 0.643 [0.632, 0.654] for valence; 0.752 [0.739, 0.765] for beauty. In the Vessel dataset (beauty only), the mean $r_{\text{MM1}}$ across respondents is 0.461 [0.408, 0.513]. These values are useful reference points in this case because they allow us to answer a number of clarifying questions about how accurate the models are, given how accurate the models could be with respect to the agreement among human respondents.

The first question we can answer with our $r_{\text{MM1}}$ values is whether higher average agreement for a given affective rating entails higher levels of predictive accuracy. Our results plainly suggest that it does not: $r_{\text{MM1}}$ values for arousal are far lower than for valence, but average predictive accuracies for arousal are slightly higher than for valence.

The second question we can answer with our $r_{\text{MM1}}$ values is how “representative” of the group-average a given model’s predicted ratings are, relative to the ratings of individual human respondents. We define “representativeness” in this case as the similarity of a reference (the group) to a comparand (an individual model or human respondent). Here, we quantify the representativeness of the perceptual models as the proportion of respondents in the human respondent pool whose $r_{\text{MM1}}$ values are lower than the value of mean predictive accuracy $r_{\left(y,\hat{y}\right)}$ across models. Using this metric, we find in the OASIS dataset that model predictions are more representative of the group-average ratings than 78%, 17%, and 74% of individual human respondents for arousal, valence, and beauty respectively. In the Vessel dataset, model predictions are more representative of the group-average ratings than 100% of human respondents (i.e. no single human respondent is as “representative” of the group as the average perceptual model). This suggests that the average object recognition model substantially exceeds the “ceiling of shared taste” (mean $r_{\text{MM1}}$) for every rating apart from valence, and yields roughly the same predictive accuracies as if we were to construct a model from the responses of the 32.5% most representative human respondents across all our affective ratings combined.

Whereas mean-minus-one correlations quantify how well an individual respondent’s ratings “predict” the group-average ratings data, the Spearman–Brown split-half reliability ($r_{\text{split}}$) quantifies how well the group-average ratings data predicts itself. In the OASIS dataset, mean $r_{\text{split}}$ values (across 10,000 splits, Spearman–Brown corrected) are 0.963 [0.945, 0.975] for arousal; 0.992 [0.990, 0.994] for valence; and 0.989 [0.984, 0.992] for beauty. In the Vessel dataset (beauty only), mean $r_{\text{split}}$ is 0.862 [0.814, 0.897].

Squaring our measure of predictive accuracy ($r_{\left(y,\hat{y}\right)}$) and dividing it by the square of this split-half reliability ($r_{\text{split}}$), we obtain our measure of explainable variance explained ($r_{\text{EVE}}^{2}$; Eq. **1**). In the OASIS dataset, mean $r_{\text{EVE}}^{2}$ across models is 0.511 [0.498, 0.524] for arousal, 0.45 [0.434, 0.465] for valence, 0.57 [0.556, 0.584] for beauty. In the Vessel dataset (beauty only), mean $r_{\text{EVE}}^{2}$ is 0.607 [0.595, 0.619]. Combined across affects (i.e., valence, arousal, and beauty), mean $r_{\text{EVE}}^{2}$ is 0.535 [0.525, 0.544].

**Fig. 2.**
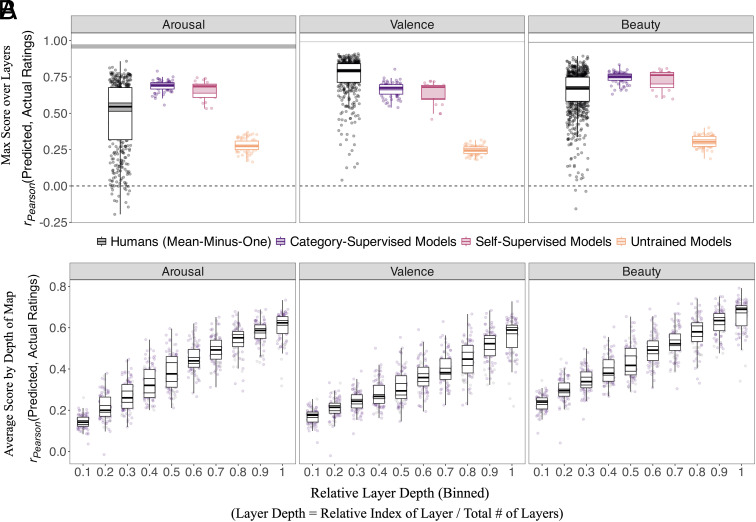
Accuracy of model-predicted affect ratings. Each colored point in these plots corresponds to an individual respondent or individual model. The gray horizontal bars are the Spearman–Brown split-half reliability noise ceilings for the group-average affect ratings, and the shaded horizontal cross-bars are the 95% bootstrapped CI of the mean across points; (*A*) shows the accuracy of the most predictive layers in each neural network model (with points in orange corresponding to untrained models). For reference, we show (in black) the mean-minus-one correlations of individual human respondents to the group average. Here, we see that the average trained model is (for arousal and beauty) about as predictive of group-average affect as the 32.5% most taste-typical human respondents, and about 70% accurate overall. Category-supervised models are no more predictive than self-supervised models, but trained models are categorically more predictive than untrained models. (*B*) shows predictive accuracies across layer depth for the trained category and self-supervised models in our survey. In this plot, the x axis is the relative depth of layer in the neural network (from 0, the earliest layer, to 1, the deepest layer), binned into slices of 10. The y axis is the average predictive accuracy in that slice, in units of *r_Pearson_*. Each point is a model trained (via category- or self-supervision) on ImageNet. Here, we see that for all 3 affect categories, the deepest layers are the most predictive, with a nearly monotonic increase in predictivity across (binned) layers.

Taken together, these results suggest that object recognition models, never trained on affect, have nonetheless learned statistical proxies of affect sufficient to predict the human affective response to images with substantial accuracy. A summary of these accuracies across models relative to our two interrater variability measures is shown in Fig. 2*A*.

#### 1.1.2. The most predictive perceptual models explain approximately 2/3 of the explainable variance in human responses.

Though the mean performance across object recognition models is informative, many of these models (e.g. VGG16) are now considered obsolete in the machine learning literature. More modern models that obtain better performance on their primary tasks (1,000-way or 21,000-way classification on ImageNet) can also provide better feature spaces for decoding affect. Here, we report the predictive accuracies for these “superlative models.” For statistical confidence, we subject the most-predictive feature spaces from each of these models to an additional bootstrapping analysis, wherein we resample the target group-average human ratings 1,000 times, and rerun the affect-decoding analysis on each of these bootstraps. The most-predictive ImageNet-1K model (a variant of Microsoft’s Swin Transformer) yields a bootstrapped $r_{\text{EVE}}^{2}$ (averaged across the 4 distinct combinations of affect category and dataset) of 0.648 [0.621, 0.672]. This same model trained on the larger ImageNet-21K benchmark yields an average $r_{\text{EVE}}^{2}$ of 0.673 [0.649, 0.695]. The most-predictive ImageNet-21K-trained model (and also the most predictive model among the N = 160 purely perceptual models) is a variant of ConvNext architecture, and yields $r_{\text{EVE}}^{2}$ of 0.729 [0.702, 0.75]. In short, the most affect-predictive purely perceptual model in our survey explains more than 2/3 of the explainable variance in the group-average human affect ratings.

### 1.2. What Kinds of Perceptual Features Are Most Predictive of Affect?

Having established the overall predictive accuracy of purely perceptual models, we can now scrutinize individual differences between models to better assess the kinds (i.e. provenance) of features that contribute to better or worse prediction.

#### 1.2.1. Trained models are categorically more predictive of affect than untrained models.

Given the size, complexity, and sometimes rich structure of feature spaces inherent to deep neural networks, there have been several cases in recent years in which randomly initialized networks—never trained—have demonstrated predictive power as robust as that of fully trained networks (59, 60). While not entirely surprising given certain architectural priors (e.g. translational invariance in convolutional neural networks), this high predictive accuracy can sometimes lead to the impression that neural networks are little more than stacks of random features (61) on which training has little impact. Here, we show to the contrary that training matters. For every ImageNet-trained model (N = 72), we compare that model’s predictive accuracy with the accuracy of its randomly initialized counterpart. To test significance, we perform pairwise *t* tests across each image category, affect rating, and dataset, with Holm corrections for multiple comparisons. We find all pairwise differences are statistically significant (at p<0.001) with often massive effect sizes (mean Hedge’s g=7.43; see Fig. 1*C*). Not a single randomly initialized model outperforms its ImageNet-trained counterpart for any affect rating (arousal, valence, or beauty).

#### 1.2.2. Representations learned for object and scene recognition are the overall best representations for predicting affect.

The results we have reported so far have been exclusive to models trained on object recognition through the ImageNet challenge. But how does object recognition fare in relation to other tasks in terms of providing features relevant for the prediction of affective processes? To answer this, we first analyze the Taskonomy models (62, 63), a set of 24 models all sharing the same base encoder architecture (ResNet50) and same input data (4.5 million indoor scenes), but each trained on 1 of 24 different canonical computer vision tasks. These tasks include variants of autoencoding, edge detection, keypoint labeling, segmentation, depth estimation, surface normal estimation, and classification, and were found by the authors of Taskonomy to cluster into one of 4 supersets (2D, 3D, geometric, and semantic tasks). In this analysis, we apply our affect decoding to each of the 24 Taskonomy models (+1 randomly initialized version of the base encoder), and rank them (as before) according to the cross-validated accuracy of their maximally predictive layers.

In all 3 affect categories of the OASIS dataset (arousal, valence, and beauty) and the single affect category of the Vessel dataset (beauty), object and scene recognition are the top 2 of the 24 (+1) task weights tested (For further details, see *SI Appendix*, section 3). To assess the reliability of these rankings, we again perform a bootstrapping analysis, resampling the human respondent pool 1,000 times and tabulating the ranks of these models for each bootstrap. For all 4 sets of affect ratings, object or scene classification was the top model in 1,000/1,000 bootstraps ($p_{\text{Holm}}<0.004$). For arousal, a nonclassification model was second in 3/1,000 bootstraps. For beauty in the Vessel dataset, a nonclassification model was second in 272/1,000 bootstraps.

While these results do indeed suggest an advantage for object and scene classification as tasks undergirding better affect prediction, it is worth noting that the raw predictive accuracies of all the Taskonomy ResNet50 models—including the object and scene classification models—are substantially lower than ResNet50 models trained on more diverse datasets (e.g. ImageNet). Importantly, this lower overall accuracy also underscores that training dataset (i.e. visual ecology or sensory experience) is another significant mediator of downstream affect prediction. (For further discussion, see Section 1.4 and *SI Appendix*, section 3.)

#### 1.2.3. The features most predictive of affect are the deepest features.

While we have so far reported only the accuracies of the single most predictive layer per model, this leaves open the question of where in the model these layers are located (i.e. how deep they are). In this analysis, we assess predictive accuracy as a function of depth in the models’ processing hierarchies. Because the majority of the models in our survey have different numbers of layers, we quantify layer depth here as a proportion: the relative position of a given layer from first to last, divided by the total number of layers, making 0 the first (shallowest) layer, and 1 the last (deepest) layer. In the OASIS dataset, the average depths (across the N = 72 ImageNet-trained models) of the most predictive layers are 0.934 [0.915, 0.953] for arousal; 0.955 [0.929, 0.98] for valence; and 0.956 [0.938, 0.974] for beauty (Fig. 1*B*). In the Vessel dataset (beauty only), the average depth is 0.887 [0.866, 0.909].

Of course, the means here do not necessarily capture what could be a multimodal distribution of highly predictive layers across the network (e.g. high predictive accuracy in early layers, low predictive accuracy in middle layers, and high predictive accuracy again in later layers). To further quantify the relationship between model layer depth (as regressor) and predictive power (as regressand), we perform a linear regression for all combinations of model, affect category, image set, and depth. In the OASIS dataset, the mean coefficients of model layer depth on overall accuracy are 0.523 [0.5, 0.546] for arousal; 0.438 [0.414, 0.462] for valence; and 0.471 [0.45, 0.493] for beauty. In the Vessel dataset (beauty only), the mean coefficient is 0.263 [0.247, 0.278]. From the first to last layer, the average increase in accuracy between predicted and actual ratings ($r_{\left(y,\hat{y}\right)}$) is 0.424 [0.408, 0.439]—a substantial effect that underscores just how much the feature hierarchy matters for predicting affect.^†^

#### 1.2.4. Self-supervised models predict affect as accurately as category-supervised models, suggesting top–down category-learning is unnecessary for accurate affect prediction.

Given the superiority of the object and scene classification models among the Taskonomy models, as well as the relative depth of the most predictive layers in the ImageNet-trained models (up to and including the softmax layers that produce category labels), one question that arises is the degree to which explicit, “top–down” feedback is necessary for accurate affect prediction. In this formulation, a model’s ability to predict affect in a given image may simply be a function of the model’s having been trained (with human labels) to distinguish identifiable, affectively charged categories (e.g. faces, weapons, or insects).

The performance of the (N = 22) self-supervised models in our survey—trained on ImageNet images, but without explicit category labels—allow us to address this in part. Averaging across all affect and image categories, the mean predictive accuracy ($r_{\left(y,\hat{y}\right)}$) of the category-supervised models is 0.685 [0.675, 0.696]; the mean of the self-supervised models is 0.688 [0.641, 0.702]. A nonparametric Mann–Whitney test shows this difference to be nonsignificant (W= 568, P= 0.687).

What about the performance of the most predictive models (including those trained on image sets larger than the 1.2 million images of ImageNet)? The most predictive object-recognition model (the ConvNext architecture trained on the 14 million images of ImageNet21K) explains 0.729 [0.702, 0.75] of the explainable variance across ratings. The most predictive self-supervised model (a variant of FaceBook’s RegNet-Self-Supervised pretraining of visual features in the wild architecture trained on 1 billion images) explains 0.688 [0.662, 0.708] of the explainable variance across ratings. Bootstrapping the difference between these models 1,000 times across the respondent pool, we find this difference to be nonsignificant (mean $\Delta r_{\left(y,\hat{y}\right)}$= 0.13 [−0.03, 0.18], p=0.64).

These results show that models trained with explicit category supervision are as predictive of affect as models trained without. At minimum, this suggests that a model’s ability to predict affect is not contingent on the kinds of explicit semantic, symbolic, or conceptual feedback inherent to “top–down” category-learning. It does not, however, preclude the possibility that this prediction is contingent on “bottom–up” implicit category-learning (i.e. invariances and selectivities learned by contrastive loss): Indeed, many of the features in adequately trained self-supervised models that support the linear decoding of affect may also support the linear decoding of category (64, 65). (For further discussion of this point, as well as baseline predictions of ratings directly from image labels, see *SI Appendix*, section 4.)

### 1.3. How Robust Is Our Prediction of Affect from Perceptual Models?

In this section, we extend the decoding paradigm to four subconditions: more directly comparing predictive accuracy across different image categories (e.g. landscapes, scenes, artworks); across the different affect categories (arousal, valence, beauty); when predicting individual respondent ratings (as opposed to group-averages); and when cross-decoding between datasets (i.e. training on the images of OASIS, and predicting affect in the images of Vessel, or vice versa). For simplicity, we report here the results only from the N = 72 ImageNet-trained models.

#### 1.3.1. There are meaningful differences in our ability to predict affect across image category: Landscapes and faces are more predictable than social scenes and art.

**Fig. 3.**
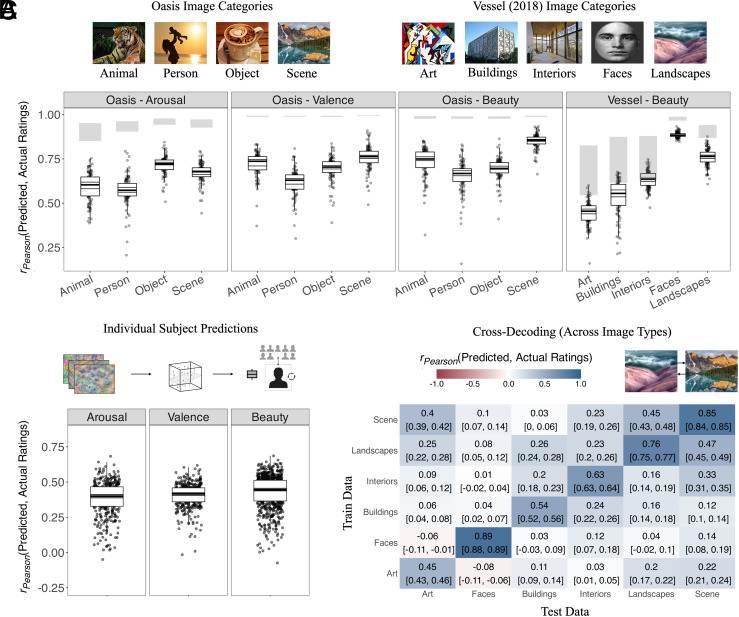
Decoding robustness across experimental subconditions. (*A*) shows the average predictive accuracy of the N = 72 ImageNet-trained models across the image (super)categories available in OASIS and Vessel datasets. The shaded rectangles in the upper half of the plot are the 95% CIs over the Spearman–Brown split-half reliability per image category. The shaded crossbars at the midpoint of the boxplots are the 95% CIs of the median model accuracy. There are large and significant differences in predictivity across image category, with Scenes and Landscapes some of the more predictable categories, and Person and Art some of the least. The overall predictivity of a category is proportional to its noise ceiling (lower noise ceiling entailing lower average accuracies; for example, in the categories of the Vessel dataset), but differences persist even when noise ceilings are relatively equal (for example, in ratings of Valence for the OASIS dataset). (*B*) shows average predictive accuracies when adapting the decoding pipeline to the ratings of individual human respondents, rather than the group average. Each point in this case is the average accuracy per human respondent across the ImageNet-trained models. While decoding fails for a nontrivial portion of the human respondents, the median predictive accuracy is relatively stable at *r_Pearson_* = 0.4 to 0.45 across respondents. (*C*) shows the results of a cross-decoding analysis between datasets, focalizing the transferability of mappings learned for group-average ratings on the subcategories of the Vessel image set to the category of Scene in the OASIS image set. In each cell is the average cross-decoding across ImageNet-trained models (with 95% bootstrapped CIs in the brackets below). On the diagonal is the original decoding accuracy when training and testing on the same image set. These results demonstrate that cross-decoding is most effective between Scenes (in Oasis) and Landscapes (in the Vessel dataset), arguably the only shared category between image sets.

So far, we have treated each of our two datasets (OASIS and the Vessel image set) as monoliths. But a more granular inspection of the (super- and sub-) categories in each dataset reveals key idiosyncrasies, particularly with respect to how “predictable” each category is.^‡^ Results from this analysis are summarized in Fig. 3*A*. Here, we report predictive accuracies ($r_{\left(y,\hat{y}\right)}$) averaged over affect and model for illustration: In the OASIS dataset (consisting of the “Scene,” “Object,” “Person” and “Animal” categories), “Scene” (the OASIS dataset’s name for landscape) is the most predictable of the image categories (with a mean $r_{\left(y,\hat{y}\right)}$ of 0.756 [0.744, 0.768]); “Person” is the least predictable of the image categories (with a mean $r_{\left(y,\hat{y}\right)}$ of 0.608 [0.596, 0.619]). In the Vessel dataset (consisting of the “Art,” “Internal Architecture,” “External Architecture,” “Faces” and “Landscape” categories), “Faces” are the most predictable of the image categories (with a mean $r_{\left(y,\hat{y}\right)}$ of 0.884 [0.882, 0.886]); “Art” is the least predictable of the image categories (with a mean $r_{\left(y,\hat{y}\right)}$ of 0.451 [0.438, 0.464]). Pairwise comparisons with Holm corrections for multiple comparisons show both of these differences to be significant, with large effect sizes ($p=4.14^{-}25$, Hedge’s g=2.34 and $p=3.26^{-}65$, Hedge’s g=10.7, respectively). In a compelling internal replication across dataset, we see no statistically significant difference between “Scene” in the OASIS dataset and “Landscape” in the Vessel dataset (p=1.0, Hedge’s g=0.0963), the only image category we might consider “common” to both.

The majority of these differences (especially in the Vessel dataset) are likely attributable to the divergent levels of interrater agreement across image category (though see *SI Appendix*, section 5 for an alternative explanation). As a measure of this divergence, we have our mean-minus-one correlations ($r_{\text{MM1}}$). Both datasets show that human respondents tend to agree on ratings of affect most substantially for landscapes. In the OASIS dataset, the mean $r_{\text{MM1}}$ values for landscapes (“Scene”) are 0.439 [0.413, 0.464] for arousal; 0.807 [0.796, 0.819] for valence; and 0.697 [0.686, 0.709] for beauty. In the Vessel dataset (beauty only), the mean $r_{\text{MM1}}$ for landscapes is 0.575 [0.511, 0.640]. In contrast, the mean $r_{\text{MM1}}$ for art (beauty only) in the Vessel dataset is 0.275 [0.206, 0.344]. In short, human respondents seem far more divided in their evaluations of art than of landscapes—a result discussed at length in previous work (53) (and also in *SI Appendix*, section 5).

#### 1.3.2. All group-average affect ratings are highly predictable; Beauty is the most predictable of all.

While the features of perceptual models do yield relatively high predictive accuracies for all three affect ratings, the overall highest accuracies we obtain are in predictions of beauty. We quantify this advantage in multiple ways, mirroring Section 1.1 above. First is the raw difference in predictive accuracies ($r_{\left(y,\hat{y}\right)}$): pairwise *t* tests (Holm-corrected) between the three kinds of affect ratings (only available in the OASIS dataset) reveal statistically significant differences in the prediction of beauty versus arousal [$t\left(138\right)=5.76,p=1.03^{-}7$, Hedge’s g=0.962] and in beauty versus valence [$t\left(140\right)=7.69,p=7.17^{-}12$, Hedge’s g=1.28]. Transforming these accuracies ($r_{\left(y,\hat{y}\right)}$) into explainable variance explained ($r_{\text{EVE}}^{2}$), so as to account for differences in the overall noise ceilings for each affect category, we compute the same pairwise *t* tests again, and show the same statistically significant differences in predicting beauty versus arousal [$t\left(139\right)=4.61,p=8.98^{-}6$, Hedge’s g=0.770] and beauty versus valence [$t\left(140\right)=9.13,p=2.09^{-}15$, Hedge’s g=1.52]. These effect size measures translate to an absolute difference in $r_{\text{EVE}}^{2}$ of 0.0589 [0.0518, 0.0658] and 0.120 [0.115, 0.125], respectively. Even controlling for differences in interrater variability, then, beauty emerges as the most predictable of the affect categories.

While the margin is small, this advantage is robust across almost all of the image categories in the OASIS dataset, as evidenced by pairwise comparisons that expand from testing difference in affect category alone to the interaction of affect and image category. There are significant differences (all p<0.001, Holm-corrected; mean Hedge’s g=1.64) in beauty versus arousal and beauty versus valence in all image categories except for Animal and Object, where only the difference in beauty versus arousal is significant.

#### 1.3.3. Regressions fit to individual human respondents show the models also predict individual affect ratings, with some exceptions.

So far, we have reported results that consist of predictions for group-average affect ratings. In the ideal, these group-averages approximate an overall central tendency of visually evoked affect. As with any average, though, they may also be masking idiosyncrasies at the level of individual human respondents. To get a better sense of how well our models might predict individual-level affect, we extracted the most performant layers from each of the ImageNet-trained models in our survey, and refit these layers to predict the affective responses of each individual human respondent. For maximum statistical power, we consider here results from only the full set of images in the OASIS image set.^§^

As for the group-level predictions, predictive accuracies for individual human respondents vary by affect category, with beauty again emerging as the most predictable affect (with a mean $r_{\left(y,\hat{y}\right)}$ of 0.43 [0.419, 0.439] across models and human respondents), followed by valence (0.404 [0.389, 0.419]), then arousal (0.381 [0.371, 0.392]). These means obscure a wide range of accuracies across human respondents. In the case of beauty, for example, the model’s predictions for the least predictable respondent were actually slightly anticorrelated with the respondent’s actual ratings, with mean accuracy ($r_{\left(y,\hat{y}\right)}$) across models of −0.08 [−0.098, −0.063]. Mean accuracy for the most predictable respondent was 0.674 [0.662, 0.685]. (See Fig. 3*B* for the distribution of accuracies over all respondents).

What factors influence how well a model is able to predict an individual human respondent? One central factor inherent to our data are the correlations between an individual human respondent’s rating and the overall group ratings – what we have so far referred to as the mean-minus-one correlations($r_{\text{MM1}}$) and what might (with some caveats) be considered a measure of “shared taste” or “taste-typicality” (54).^¶^ To assess the relationship between a respondent’s $r_{\text{MM1}}$ and the accuracy of a model in predicting that respondent’s ratings, we first average the predictive accuracies per respondent across model. We then correlate each respondent’s $r_{\text{MM1}}$ with this average. Across all three affect ratings, we find the relationship between a respondent’s $r_{\text{MM1}}$ and their mean $r_{\left(y,\hat{y}\right)}$ across models to be statistically significant: with correlations of 0.359 [0.349, 0.368] for arousal, 0.489 [0.471, 0.508] for valence, and (most substantially) 0.679 [0.669, 0.689] for beauty.

It is worth emphasizing here that this relationship is not accounted for by differences in the number of images that the respondents viewed (all respondents viewed between 223 and 225 images), nor in the particular kinds of images they viewed (since each respondent’s mean-minus-one correlation is calculated only on the subset of images that particular respondent viewed). Thus, the statistical power to learn each respondent’s ratings function is constant across regressions. All that differs is that function’s learnability: The more “divergent” a respondent’s response profile, the less predictable that respondent’s ratings will be on the basis of the features yielded by the models. (For a more extended discussion and interpretation of this result, see *SI Appendix*, section 6.)

#### 1.3.4. Cross-decoding: Regressions fit to one image set can often predict the other.

So far, the results we have presented here are based on the leave-one-out cross-validated predictive accuracies computed from the regressions fit to each image set. A more stringent test of generalizability is whether regressions fit to the beauty ratings of one image set can predict beauty ratings in the other. To run this test, we took the most predictive feature spaces from each of our surveyed models, and used them as a common set of predictors for a series of regressions we iteratively fit to one image subset, then tested on another. Each of these iterations produces a “cross-decoding accuracy:” the correlation between the predicted and actual ratings of the held out (test) image set when fitting on the target. With all iterations complete, we obtain what is effectively a distance matrix, wherein each cell contains the cross-decoding accuracy between categories (For an example of a subset of this correlation matrix, see Fig. 3*C*).

While each cell of this cross-decoding matrix is a result in and of itself, the main takeaway from the matrix in its aggregate is that where we would reasonably expect to find high cross-decoding accuracies, we frequently do find high cross-decoding accuracies. For example, let us consider the “landscape” category (scenes)—the only image category directly shared across the image sets. Training on landscapes from the OASIS image set produces an average cross-decoding accuracy of 0.45 on landscapes from the Vessel image set; vice versa, training on landscapes from the Vessel image set produces an average cross-decoding accuracy of 0.47 on the OASIS image set. Though these accuracies are slightly lower than the ones we obtain when fitting regressions within image set, their similarities suggest that distributional differences between the image sets are not so substantial as to prevent generalization between image set—at least for landscapes. Intuitively, features relevant for other (more specific) image categories, like the art and faces of the Vessel dataset, consistently fail to generalize: features fit to the more general category of landscapes from the Vessel dataset, for example, yield an average cross-decoding accuracy of 0.311 [0.294, 0.329] across the image categories of the OASIS image set; features fit to the more limited category of faces yield an average of 0.055 [0.038, 0.072] (see *SI Appendix*, Fig. 2, section 9).

### 1.4. Beyond Pure Perception: Exploring the Predictivity of Hybrid Vision-Language Models.

The single most predictive purely perceptual model (out of the total 160) in our survey yields a bootstrapped average explainable variance explained ($r_{\text{EVE}}^{2}$) of 0.729 [0.702, 0.75]. What factors might account for the approximate quarter (∼0.271) of explainable variance this model fails to explain? One candidate is language, which we often use to conceptualize and communicate the affect we feel in response to things we see. In this section, we explore the affect predictions of recently developed vision-language models that learn representations from visual and linguistic input simultaneously.

#### 1.4.1. CLIP-style language-aligned vision models explain more variance than pure vision models.

Perhaps the preeminent example of recent developments in hybrid vision-language deep learning is OpenAI’s Contrastive Language-Image Pretraining (CLIP) (66), a “multimodal” deep neural network model that learns to match latent representations of a given image with a textual caption that accompanies the image. CLIP consists of a visual encoder (either a vision transformer or a ResNet 50/101 architecture) and a language encoder (typically a BERT transformer). These encoders are trained simultaneously via a contrastive loss that pits the latent space embeddings of the encoded textual caption against the latent space of the encoded image. This training procedure is notable for producing representations that are both more robust to the kinds of adversarial perturbations that typically degrade the performance of supervised models (67) and are abstract enough, for example, to equate a photograph and cartoon of the same visual concept (68)—much like the famous “gnostic” neurons found in the human temporal lobe (69).

Decoding ratings of affect from the visual encoders of 6 OpenAI-CLIP models, we find the representations learned by these models partially close the gap on the variance left unexplained by the purely perceptual models. The best performing CLIP model (ViT-Large-Patch14) yields a bootstrapped average $r_{\text{EVE}}^{2}$ of 0.870 [0.857, 0.881] across the 4 combinations of affect category and dataset, versus ConvNext-Large’s average of 0.729 [0.702, 0.75]. Honing in on individual combinations of image and affect category, we find particularly strong gains in the Vessel image set. The most predictive CLIP model, for example, yields $r_{\text{EVE}}^{2}$ of 0.924 [0.637, 1.195] for ratings of the art category. The most predictive ImageNet-trained model (ConvNext-Base) yields $r_{\text{EVE}}^{2}$ of 0.617 [0.253, 0.977] for this same category.

#### 1.4.2. Controlling for dataset and architecture, language-aligned vision models still show an advantage over pure vision models.

A major caveat to the OpenAI-CLIP results is a certain level of ambiguity as to which exact aspect of the CLIP training procedure underlies the gains in predictivity. While the abstractions encouraged by the natural language contrast may seem like the prime candidate (since the underlying network architectures are relatively standard), CLIP models are trained on 400 million text-image pairs. ImageNet-trained models are trained on 1.1 million images. This raises the possibility that CLIP’s gains are merely a function of the increased number (or diversity) of training samples (*SI Appendix*, section 7).

A more controlled test for the role of language in predicting affect is available in the form of FaceBook’s Self-supervision meets Language-Image Pretraining (SLIP) models (70): a collection of 9 models, built with 1 of 3 architectures (ViT-Small, Base, and Large), and 1 of 3 optimization targets (SIMCLR-style purely visual self-supervision, CLIP-style language-supervision alone, and a combination of the two—the eponymous SLIP), all trained on the same image set (YFCC15M). Comparing across these models allows us to better isolate the effect of language from the effects of architecture and input (visual ecology). Bootstrapped pairwise comparisons between these models demonstrate that pure self-supervision (SimCLR) performs significantly worse than pure language-supervision (CLIP) in both the Small and Large ViT architectures for 1,000/1,000 bootstraps ($p_{\text{Holm}}$= 0.009, in both cases), but by diminishingly small margins in both cases (with differences in $r_{\text{EVE}}^{2}$ of 0.041 [0.026, 0.056], and 0.046 [0.028, 0.062], respectively). CLIP does not significantly outperform SimCLR in the Base ViT Architecture (with differences greater than 0 in only 885/1,000 bootstraps ($p_{\text{Holm}}$= 1.0), and yields average gains in $r_{\text{EVE}}^{2}$ of −0.009 [−0.025, 0.008]). SLIP (the model that combines self- and language-supervision) outperforms CLIP and SimCLR for all architectures ($p_{\text{Holm}}$= 0.009, in all cases), but does so most substantially in the ViT-Large architecture (with an average gain in $r_{\text{EVE}}^{2}$ 0.121 [0.134, 0.106] over CLIP alone). These results are a point in favor of language as a significant factor in building representations that are more predictive of affect, but still do not fully account for the gains of OpenAI’s CLIP, whose ViT-Large outperforms SLIP’s ViT-Large by another average gain of 0.121 [0.134, 0.106]—a strong indication that visual ecology (i.e. the images used to pretrain the model) still matters.

Importantly, we cannot consider hybrid-vision language models like CLIP and SLIP to be “purely perceptual models.” Though the visual encoders of these models operate at a computational level of description almost identical to that of the other models in our survey (a series of nonlinear, feedforward transformations of input pixels into a lower-dimension latent space), the language-contrastive learning target connotes a major algorithmic divergence. While recent work has begun scrutinizing how representation in the most anterior stages of the ventral visual processing pathway may be “aligned” to language in much the same way CLIP visual encoder is constrained to align its representations with those of a language encoder (71), the influence of linguistic abstraction on perceptual processing largely remains a mystery. On a more practical level, the training of CLIP on largely unfiltered image-text pairs means that affective information may well be implicit in the optimization function in a way it demonstrably is not for the other models in our survey. Consider the case in which a CLIP model learns to align the textual caption, “a picture of a beautiful sunset,” with an image of a sunset. The presence of affective labels in the captions could feasibly facilitate downstream decoding. OpenAI has so far declined to publicly release the dataset on which CLIP was trained, but it is reasonable to assume these kinds of labels are present in a statistically nontrivial portion of the training set given the preponderance of affective tags on publicly posted online images.

The various caveats and considerations that accompany the testing of CLIP and SLIP models mean we cannot yet confidently interpret their superiority as direct evidence that the abstractions of language are necessary to account for the variance unexplained by our purely perceptual models. However, the performance of these models does suggest a concrete target for future empirical investigation, in which self-supervised models trained on much larger image sets are pitted directly against models trained with CLIP’s linguistic alignment target, and ideally also trained with further controls that account for the presence of explicitly affective language in the textual captions.

## 2. Discussion

The experience of visually evoked affect (“seeing with feeling”) is a near-universal phenomenon without a universal definition, and one whose provenance remains a matter of hotly contested debate (3, 9, 72–80). In many ways, the nature of this debate is mereological: a question of how each constituent part of our visual affective experiences (i.e. physiology, perception, cognition) defines the whole (i.e. the “feeling” itself). As humans, we cannot help but experience this whole—and as scientists of human behavior, we cannot decompose the whole back into its constituent parts to better understand its composition. But we can circumvent this dilemma (at least partially) by turning to machines—whose experiences are the product of engineering choices, and by extension, of choices within our experimental control. In this work, we demonstrate how the emergent variation in a large survey of industry-standard machine vision models allows us to answer two key questions about the computational mereology of visually evoked affect: Namely, how much of that affect can be predicted by perceptual computations alone, and which key ingredients of these computations allow us to make those predictions as accurately as we do.

The various analyses of this work suggest the answer to the first question (“how much...”) is that perceptual computations alone can predict a substantial majority of the average human affective response to images. The answer to the second question (“with which ingredients...”) is that the key to this prediction is not just perception per se (i.e. the translation of a given sensory input into units of behaviorally relevant “meaning”), but representation learning: the construction of the units of meaning by way of repeated experience with the patterns that define the sensory world. To better grasp why this set of findings matters for our understanding of the relationship between perception and affect more generally, we expand on both of these answers below.

Previous work has already established that machine vision systems are capable of predicting a wide range of “higher-order” concepts (and associated behaviors) that many once considered “beyond the image” (21, 22, 24, 29, 81, 82), including (most relevantly) the prediction of affect in OASIS images (75) and the binary classification of art versus nonart images (18). Yet, these studies (which focus on pixel-computable image statistics; features extracted from single, simpler Deep Neural Network (DNN) models; or DNN models trained end-to-end to predict the concepts themselves) did not fully establish just how much of these “higher-order” phenomena we could (in practice) predict—that is, the maximum amount of variance these machines could explain when assessed at their empirical limits. Without this upper bound, one could easily make the argument that “higher-order” processes (e.g. cognitive interpretation) might still be the main or dominant determinants of “higher-order” phenomena (e.g. affect). But if perceptual computations alone can predict the majority of explainable variance in “higher-order” behavioral response patterns, this argument seems quite a bit less tenable.

More important than the raw point values of these predictions, however, is the motivation they give us to more deeply interrogate how or even why perceptual models could predict affect so accurately in the first place. Having representational models as performant as modern deep neural networks at our empirical disposal is a relatively recent phenomenon. As such, theories that specifically account for the behavior of these models are still somewhat nascent. But theories preceding the advent of these models do suggest a few reasonable starting points for further understanding in the future. Theories of predictive processing, for example, suggest these models may in effect be placeholders for principled instantiations of perceptual or representational “priors” (83–85), and that many forms of visually evoked affect may in large part track with learning from the perceptual “surprise” that comes with (learnable) deviation from these priors (86–89). Another set of theories, predicated on seminal work in “affective prediction” (3) and “microvalences” (90), suggests that the perceptual models we survey may contain statistical proxies of the downstream value we can extract from the objects and affordances of our physical environment. Yet another possibility, predicated on the computational and theoretical neuroscience literatures, is that the perceptual models we survey may serve as reasonable approximations of systems (like many biological perceptual systems) that encourage or actively enforce some form of representational sparsity (91–94).

Our own interpretation of the success of purely perceptual models in the prediction of affect is that these models are defined by their ability to learn—and in particular, to learn behaviorally relevant hierarchical structure directly from the statistics of naturalistic input that we can derive from the same kinds of sensory ecologies inhabited by human observers. Affect will be fundamentally yoked to this learning not because affect is inherent to any one feedforward pass of the perceptual cascade, but because affect provides a compass both for learning that has already occurred and for learning yet to come—whether that learning be of categories, of affordances, or even of epistemics. [Theories of aesthetics, in particular, stress that something like this may be a primary reason we experience beauty in the first place (74, 95–101)].

This interpretation is supported by a number of the findings in this work: Randomly initialized perceptual models, for example, explain only a small fraction of the variance that fully trained models explain. Models trained on datasets that do not fully reflect the full statistics of natural images (i.e. the Taskonomy models) are more predictive than randomly initialized models, but not nearly as predictive as models trained on more diverse datasets (such as ImageNet). The success of OpenAI’s language-aligned CLIP model relative to other language-aligned models seems also to be a function (in no small part) of its more extensive training data. Our emphasis on experience here is intentional; while modern machine vision machine systems can and should be considered image-computable models, their ability to predict higher-order image properties is largely not a function of their ability to extract information from any given single image, but is instead a function of their ability to learn where (with respect to the representation of previously seen images) a newly seen image should be routed. Without learning from the statistics of experience with the same kinds of visual stimuli we regularly encounter as humans, these models lose the vast majority of their affect-predictive power.

Zooming out, then, if we were to situate the variance in affective experience explained by perceptual machines along an axis from input (e.g. pixels) to ideation (e.g. judgment), that variance would likely fall somewhere in the middle—in line with brain imaging work that finds correlates of affective and aesthetic value in or near high-level visual regions (74, 102–105) and behavioral work that favors hierarchical structure over low-level visual properties in predicting judgments of naturalistic stimuli (106, 107). The statistical proxies of visually evoked affect inherent to our perceptual machines are statistical proxies circumscribed by input, but shaped by representational priority. These proxies by their nature will be idiosyncratic to the agent who relies on them to calibrate behavior. This axis, we should note, runs parallel to well-established axes in empirical aesthetics like “formalism versus contextualism” (108, 109) and has its natural endpoints in work that focalizes raw images on one end, and work that focalizes pure cognition on the other (50). In between, we find work that focalizes everything from genetics (110) to cultural vogue and social capital—and work like ours (95, 111), which focalizes perceptual hierarchies.

Note that while this “resituating” of the computational basis for “seeing with feeling” may be a story largely familiar to those more steeped in the science of aesthetics, somewhat latent across our analyses is another result that may come as a bit of surprise to those more steeped in affective science: that is, the difference (or lack thereof) in the ability of perceptual models to predict arousal, valence, and beauty alike. This finding partly challenges a conceptual hierarchy of affect in which physiologically grounded “core affect” (valence and arousal) (4) may be felt even in the absence of higher-order “conceptual knowledge” (112) and is in this sense distinct from more cognitively complex forms of affect and emotion (e.g. beauty) that seem to require conceptual “thought” (50, 113). The finding that purely perceptual models predict both core affect and beauty suggests this theorized hierarchy (of affective experience) might ultimately be flatter than it initially appears.

Future work, we hope, will further resolve what exactly our decoders seem sensitive enough to detect, and in so doing will further demystify the experience of visually evoked affect with all the power and precision of the experimental tools researchers have developed in the search to demystify perception. The meteoric rise of perceptual machines may be a meaningful next step along the road to a computationally principled, mechanistic, stimulus-computable account of what it means to “see” with “feeling,” but many more steps will be necessary to see, feel, and understand all together.

## 3. Materials and Methods

### 3.1. Image Datasets.

As our primary dataset, we use OASIS (51), a set of 900 images spanning four distinct categories (people, animals, objects, and scenes), with normed ratings of arousal and valence from 822 human respondents. Ratings of beauty (from another 751 human respondents) were obtained from an auxiliary source (52). In this work, we complement OASIS with a secondary dataset consisting of 512 images across five distinct categories (art, faces, landscapes, internal, and external architecture) (53), but for which only ratings of beauty (“aesthetic appeal”) are available. This secondary dataset allows us not only to explore various questions that OASIS does not (e.g. judgments of art versus judgments of natural scenes) but to internally replicate at least a subset of the results we obtain with OASIS.

We calculate two forms of reliability as gauges for the comparative performance of our models. The first—“mean-minus-one” reliability (53)—involves iteratively removing one respondent from the respondent pool and correlating that respondent’s ratings with the average ratings of the respondents remaining ($r_{\text{MM1}}$). The 95% CI over these leave-one-out correlations for all respondents gives us a sense of how well, on average, a randomly selected human respondent is able to predict the mean rating for a given set of stimuli (a “shared taste” ceiling). Our second reliability metric—the split-half reliability ($r_{\text{split}}$)—involves splitting the group-level data in half 10,000 times and correlating each half with the other. The 95% CI over these split-halves (corrected with the Spearman–Brown prophesy formula) provides a more concrete upper bound (a noise ceiling) on how well any predictive model could do in predicting the mean rating for a given set of stimuli. We use both of these thresholds as a point of reference for the performance of our models.

Further details about dataset collection, including the prompts used to solicit affective ratings and reliability calculations may be found in *SI Appendix*, section 1.

### 3.2. Deep Neural Network (Feature) Models.

In total, we survey a set of 180 distinct models (252 including the randomly initialized versions of a designated subset). These models are sourced from six different repositories: the Torchvision (PyTorch) model zoo (114); the pytorch-image-models (timm) library (115); the Visual Self-Supervised Learning (Library) (self-supervised) model zoo (116); the Taskonomy (visualpriors) project (62, 63, 117); OpenAI’s CLIP repository (66); and FaceBook’s SLIP repository (70). See *SI Appendix* for full descriptions of the models.

### 3.3. Decoding Method: Feature Regression.

To predict ratings of arousal, valence, and beauty for each of the images in our datasets from a given set of deep net features, we use regularized linear regression with cross-validation. Our feature regression pipeline consists of 4 distinct phases: feature extraction; dimensionality reduction; ridge regression; cross-validation and scoring. A schematic of this pipeline is available in Fig. 1*A*.

#### 3.3.1. Feature extraction.

We consider feature extraction from “every layer” to mean the sampling of network activity generated after each distinct computational suboperation in a deep neural network model. This means, for example, that we consider a convolution and the nonlinearity that follows it as two distinct operations that produce two distinct feature spaces, both of which we consider candidates for decoding. If a layer returns a tensor with multiple components (such as a convolutional layer) we first flatten the tensor to a single component, such that the layer represents any given image as a feature vector. The layer thus represents a dataset of n images as an array $F\in R^{n\times D}$, where D is the dimensions of the feature vector.

#### 3.3.2. Sparse random projection.

For some deep-net layers D is very large, and as such performing ridge regression directly on F is prohibitively expensive, with at best linear complexity with D, $O(n^{2}D)$ (121). Fortunately it follows from the Johnson–Lindenstrauss lemma (122, 123) that F can be projected down to a low-dimensional embedding $P\in R^{n\times p}$ that preserves pair-wise distances of points in F with errors bounded by a factor ϵ. If u and v are any two feature vectors from F, and $u_{p}$ and $v_{p}$ are the low-dimensional projected vectors, then;

[2] $$\left(1-\epsilon \right)||u-{v||}^{2}<||u_{p}-v_{p}{\left|\right|}^{2}<\left(1+\epsilon \right)||u-{v||}^{2}$$

Eq. **2** holds provided that $p\geq \frac{4\ln(n)}{\epsilon^{2}/2-\epsilon^{3}/3}$ (124). With N = 900 for our dataset, to preserve distances with a distortion factor of ϵ=0.1 requires ≥5,830 dimensions. Thus we chose to project F to $P\in R^{n\times 5,830}$ in instances where D>>5,830. To find the mapping from F to P we used sparse random projections following (125). The authors show a P satisfying Eq. **2** can be found by P=FR, where R is a sparse, n×p matrix, with i.i.d elements

[3] $$\begin{array}{c} r_{\mathrm{ji}}=\left\{\begin{array}{cc} \sqrt{\frac{\sqrt{D}}{p}} & \text{with prob.}\frac{1}{2\sqrt{D}}\\ 0 & \text{with prob.}1-\frac{1}{\sqrt{D}}\\ -\sqrt{\frac{\sqrt{D}}{p}} & \text{with prob.}\frac{1}{2\sqrt{D}} \end{array}\right. \end{array}$$

$r_{\mathrm{ji}}$ in this case refers to the “random projection” (j) per feature (i). [Note that this equation is only a slight reworking of the definition provided by Scikit-Learn (126).]

#### 3.3.3. Ridge regression with LOOCV.

We used regularized ridge regression to linearly decode ratings of affect, Y, from candidate (dimensionality-reduced) deep neural network features, P. As our goal was not to identify a particular regression model for later use, but rather to obtain a best estimate of decoding (predictive) accuracy from candidate feature spaces, we utilized all the data at our disposal with a leave-one-out (generalized) cross-validation procedure. For every image in our dataset (∀i∈{1…900}) we fit the coefficients ${\hat{\beta }}_{i}$ of a regression model on the remaining data, such that $Y_{-i}=P_{-i}\hat{\beta_{i}}+\epsilon$ with minimal ‖ϵ‖ (error). Ridge regression penalizes large $\|\hat{\beta }\|$ proportional to a hyperparameter λ, which is useful to prevent overfitting when regressors are high-dimensional (as with P). We first standardized Y and the columns of P to have a mean of 0 and SD of 1. Let $P_{-i}$ and $Y_{-i}$ denote P and Y with row i missing, then each ${\hat{\beta }}_{i}$ is calculated by;

[4] $${\hat{\beta }}_{i}={\left(P_{-i}^{\prime }P_{-i}+\lambda I_{p}\right)}^{-1}P_{-i}^{\prime }Y_{-i}$$

Each $\hat{\beta_{i}}$ is then used to predict the beauty rating from the deep-net feature projection of each left-out image;

[5] $${\hat{y}}_{i}=P_{i}\hat{\beta_{i}},\;\;\;\hat{Y}={\left\{{\hat{y}}_{i}\right\}}_{i=1}^{900}$$

The hyperparameter λ we set at 1e4, a value we determined using a logarithmic grid search over 1e−1 to 1e6 on an AlexNet model that we subsequently exclude from the main analysis. λ=1e4 yielded the smallest cross-validated error ($\|Y-\hat{Y}\|$) when averaging across layers. We used the RidgeCV function from ref. 126 to implement this cross-validated ridge regression, as its matrix algebraic implementation identifies each $\hat{\beta_{i}}$ in parallel, resulting in significant speedups (127).

#### 3.3.4. Scoring.

In this analysis, we “score” each deep-net layer by computing the Pearson correlation coefficient between predicted ratings, $\hat{Y}$, and the actual group-average affect ratings from the human respondents, Y—a metric we refer to as $r_{\left(y,\hat{y}\right)}$. To convert this Pearson correlation coefficient into a score that represents the proportion of “explainable variance explained,” we divide the square of this coefficient by the square of the Spearman–Brown split-half reliability that constitutes the noise ceiling—a metric we refer to as $r_{\text{EVE}}^{2}$.

## Supplementary Material

Appendix 01 (PDF)

## Data Availability

Previously published data were used for this work (51–53).

## Footnotes

^*^Note that our justification in treating “category-supervised” object recognition models as purely perceptual models is somewhat historical, and a more modern theoretical understanding might instead favor “self-supervised models” as a more justifiable choice for the label of “purely perceptual.” For further discussion of this point, see Section 1.2.

^†^We can, for a more standard measure of effect size, convert our regression coefficients to standardized betas (β). In the OASIS dataset, β= 0.881 [0.85, 0.911] for arousal; 0.825 [0.793, 0.857] for valence; 0.852 [0.821, 0.884] for beauty. In the Vessel dataset (beauty only), β= 0.749 [0.708, 0.79].

^‡^Note: These categories are categories provided by the authors of the original datasets. We made no modifications to any category from either dataset.

^§^Human respondents in the Vessel experiments rated only certain subsets (categories) of images, making an analysis of individual ratings for the combined images impracticable.

^¶^The most immediate caveat here is the absence of better measures of intrarater reliability; in other words, how consistent across time the human respondents are in their individual ratings. In the absence of this measure, it is unclear whether an individual respondent’s taste-atypicality is indicative of systematic divergence from the group-average or erratic behavior at the time of assessment.
