# Supplementary material for: The perceptual primacy of feeling: Affectless visual machines explain a majority of variance in human visually evoked affect
Source: Proc Natl Acad Sci U S A. 2025 Jan 23;122(4):e2306025121. doi: 10.1073/pnas.2306025121 (PMC11789064; doi:10.1073/pnas.2306025121)
Supplement: Supplementary file 1 — Appendix 01 (PDF) [file pnas.2306025121.sapp.pdf]

The Perceptual Primacy of Feeling:
Supplementary Information

1 Glossary of Notations and Acronyms (Metrics, Datasets, Models)

Below, we provide the expanded form, definition, and/or reference for the various acronyms found throughout the main body of the manuscript and the supplementary material.

Metric Acronyms

- r_{split}: *Spearman-Brown split-half reliability* (noise ceiling of the human behavioral data); the mean Pearson correlation between 1000 random splits of the target response dataset.
- r^2_{split}: *squared Spearman-Brown split-half reliability*; literally, the square of the mean Pearson correlation between 1000 random splits of the target response dataset.
- r_{MM1}: *mean-minus-one correlation* ('ceiling of shared taste'); the mean Pearson correlation value obtained when correlating the responses of a single, held-out subject with all other subjects in the target response dataset.
- $r_{(y,\hat{y})}$: *correlation between predicted and actual values* (a measure of predictive accuracy); in this work, the Pearson correlation between a model's predicted response data and the ground-truth response data.
- r^2_{EVE}: *explainable variance explained* (a measure of predictive accuracy, normalized by the 'noise ceiling' of the data to be predicted); in this work, the squared Pearson correlation between the predicted and actual response data ($r_{(y,\hat{y})}$), divided by the squared Spearman-Brown split-half reliability (r^2_{split}).

Dataset Acronyms

- **AVA**: ***A****esthetic* ***V****isual* ***A****nalysis*; 'a large-scale database for aesthetic visual analysis' (1), often used (in other works) for end-to-end training of specialist aesthetic models.
- **OASIS**: *the Open Affective Standardized Image Set*; in this work, the main source of human affect ratings of arousal, valence (2) and beauty (3).

Model Acronyms

- **AlexNet**: a convolutional neural network architecture eponymously named for the lead author of the work that introduced it (Alex Krizhevsky) (4), often considered the seminal model of the modern deep learning revival.
- **VGG**: ***V****isual* ***G****eometry* ***G****roup* (5); an early family of neural network architectures, remarkable in practice as much for the width of its convolutional feature maps, as much as its once state-of-the-art depth.
- **ResNet**: ***Res****idual* ***Net****work* (6); a common convolutional neural network model or submodule, named for its use of 'skip connections' to allow better flow of gradients (loss) between deep net layers.
- **ViT**: ***Vi****sion* ***T****ransformer* (7); a variant of the transformer neural network architecture (originally developed for natural language processing), redesigned for use in machine vision. The primary innovation of vision transformers was the total substitution of all convolutional operations in favor of attention heads applied to images parcelled into patches.
- **SWin**: ***S****hifted* ***Win****dows Transformer* (8): a modified vision transformer designed to augment otherwise standard stacked attention heads with operations that more efficiently handle varying image scale and resolution.

- **ConvNext**: *"A ConvNet for the 2020s"* (9): An updated variant of a convolutional neural architecture designed to mimic the 'advantages' of vision transformers without the use of attention heads. Like SWin (a vision transformer designed to mimic key advantages of convolutional models), ConvNext (a convolutional model designed to mimic key advantages of vision transformers) is a model designed explicitly to test claims of the advantage attributable to different architectural inductive biases.
- **SimCLR**: *"A **Sim**ple Framework for **C**ontrastive **L**earning of Visual **R**epresentations"* (10); self-supervised model(s) trained only on data-implicit labels of "same" or "different" (positive and negative samples), with large batch-sizes helping to minimize the possibility of the once frequent failure mode (mode collapse).
- **SEER**: *'**Se**lf-Sup**er**vised pretraining of visual features in the wild*: A purely visual self-supervised model trained on a large natural image dataset of 1 billion random images.
- **DINO**: a self-supervised learning method ('self-distillation') designed to maximize the downstream utility of pretrained vision transformers in both dense (i.e. segmentation) and sparse (categorization) recognition tasks (11).
- **CLIP**: ***C**ontrastive **L**anguage-**I**mage **P**retraining* (12); a variant of multimodal self-supervised learning, sometimes referred to as "natural-language"-supervised learning, CLIP learns semantically rich visual representations by learning to match the latent embedding of an image to the latent embedding (from a vision encoder) of an image caption (from a text encoder). In most cases (including in this work), only the pretrained visual encoder is used in downstream tasks.
- **SLIP**: *'**S**elf-supervision meets **l**anguage-**i**mage **p**retraining"* (13); a multi-modal self-supervised learning model that combines the visual augmentation regime of SimCLR-style self-supervised learning with the language-alignment learning of CLIP. Occasionally, in this work and elsewhere, the SLIP acronym is also used in reference to the SLIP 'model family' or 'model suite' – which provides a model-based 'opportunistic experiment' (**?**) allowing for the controlled comparison of purely visual contrastive learning (SimCLR) with language-image pretraining (CLIP), holding dataset and architecture constant.
- **VISSL**: **V**isual **S**elf-**S**upervised **L**earning (Library) (14); a diverse and comprehensive model zoo of pretrained, self-supervised vision models, maintained through early 2023.

2 Human Behavioral Paradigm Details

OASIS ratings were provided by 409 human respondents for arousal, 413 for valence, and 757 for aesthetics. Each human respondent in the behavioral paradigm for the collection of the original OASIS ratings (arousal and valence) (2) was tasked with rating 225 images, sampled from across 4 image categories: Animals, Object, Scenes, People. This collection procedure was mirrored in subsequent collections of aesthetic ratings for OASIS images (15), though the prompt used to solicit ratings was altered slightly for the target affect.

The prompts given to the human participants are reproduced verbatim in Table 1 below.

Ratings for the Vessel dataset (beauty only) were provided by 24 participants for the 'visual art' image set, 17 for the 'interior architecture' image set, 19 for the 'exterior architecture' image set, 18 for the landscapes image set, and 20 for the 'faces' image set.

While the exact prompt varied slightly by image set, the general set of instructions used to solicit ratings for the 'exterior architecture' image set are reproduced verbatim below.

> Participants were instructed to make judgments based on how much they, individually, were "moved" by the images, and that they should imagine that their evaluations of the images would be used to help curate a collection of images in the given domain. In all cases, participants were told that there were no right or wrong answers. As an example, the instructions for the architecture group stated: Imagine that the images you see are of structures that may be featured in a book on architecture. The editor needs to know which structures are the most aesthetically pleasing based on how strongly you as an individual respond to them. Your job is to give your gut-level response, based on how much you find the structure beautiful,

Table 1: Prompts used in the collection of affect ratings; phrasing of *Arousal* and *Valence* is from Kurdi et al. (2); phrasing of *Beauty* is from Brielmann and Pelli (15)

Screen	Valence	Arousal	Beauty
1	Pictures communicate. They depict people, objects, and scenes. In this study, we are interested in how various pictures make people feel. As you answer, please remember: There are no right or wrong answers. We are interested in your individual opinion. Please do not take too long over any picture. Your first response is as good as any.		
2	We will ask you to rate a series of pictures in terms of how positive or negative they are. If the picture represents something good or positive, please use the right side of the scale to mark your answer. Positive images are those that represent things that make us happy, satisfied, competent, proud, contented, delighted, and so on. It doesn't matter what the specific picture is about, as long as it represents something positive or good. If the picture represents something bad or negative, please use the left side of the scale to mark your answer. Negative images are those that represent things that make us unhappy, upset, irritated, angry, sad, depressed, and so on. It doesn't matter what the specific picture is about, as long as it is something negative or bad. Use the middle of the scale to indicate that the picture makes you feel neutral, that is, that you think that the picture is neither positive nor negative. Please use the full range of the scale to make your responses rather than relying on only a few points.	We will ask you to rate a series of pictures in terms of the amount of emotion that they evoke. In other words, we would like to know how much emotional intensity the picture creates; whether the picture captures something good or bad doesn't matter. We are interested only in the degree of excitement, energy, or intensity of feeling it represents. Use the right side of the scale to mark your answer if the picture represents something that is strongly emotional. The words that we might use to describe the emotional state that the picture creates are aroused, alert, activated, charged, or energized. Use the left side of the scale to mark your answer if the picture represents something that is not strongly emotional. The words that we might use to describe the emotional state that the picture creates are unaroused, slow, still, de-energized, calm, or peaceful. Use the middle of the scale to indicate an image that is moderately arousing or halfway through the two extremes. Please use the full range of the scale to make your responses rather than relying on only a few points.	We will ask you to rate a series of pictures in terms of how much beauty they make you feel. In other words, we would like to know how intense the feeling of beauty is that the picture evokes; whether it is good or bad doesn't matter. Use the right side (bottom on a mobile device) of the scale to mark your answer if the picture makes you feel intense beauty. Use the left side (top on a mobile device) of the scale to mark your answer if the picture makes you feel no beauty at all. Use the middle of the scale to indicate that you are feeling moderate beauty, that is, halfway through the two extremes. Please use the full range of the scale to make your responses rather than relying on only a few points.
3		When making your ratings, you need to forget about whether the picture is about something positive or negative, good or bad. Instead, we ask you to rate the picture only in terms of the emotional intensity that it evokes.<br><br>For instance, you might have two pictures—one depicting an athlete winning a gold medal (something good) and another depicting an athlete getting injured and losing the race (something bad). You can give both images the same rating because they are both creating a similar level of emotion, even though the feelings are not the same. Your answers should be based only on how much emotional intensity the picture captures.	When making your ratings, you need to forget about whether the picture depicts something good or bad. We ask you to rate the picture only in terms of the intensity of beauty it makes you feel and not in terms of goodness or badness.<br><br>For instance, you might have two pictures—one depicting a landscape on a sunny summer day (something good) and another depicting a landscape during an autumn thunderstorm (something bad). You can give both images the same rating because they are both creating a similar level of beauty, even though the feelings are not the same. Your answers should be based only on how strong the feeling of beauty is the picture creates.

compelling, or powerful. Note: The structures may cover the entire range from "beautiful" to "strange" or even "ugly." We ask that you respond on the basis of how much this structure "moves" you. What is most important is for you to indicate which structures you find powerful, pleasing, or profound.

3 Model Selection Details

Further details on the models provided by each of the major model repositories contributing to our candidate model survey may be found in the list below:

- The Torchvision and Timm repositories offer pretrained versions of a large number of object recognition models with varying kinds of architectures: convolutional networks, vision transformers, normalization-free networks, and MLP-Mixer models, all trained on the ImageNet-1K classification challenge. Also included are a smaller subset of models (mostly vision transformers) trained on the ImageNet-21K classification challenge, which involves

training on a larger number of images (14.3 million versus 1.2 million) and a larger number of categories (21000 versus 1000).

- For each of the models from the Torchvision and Timm repositories, we include one trained and one randomly initialized variant (using whatever initialization scheme the model authors deem best) to assess in the aggregate whether or not training augments predictive power.
- The VISSL self-supervised models are mainly variants on a popular convolutional architecture (ResNet50), though do include the transformers of DINO (11) and the RegNet architectures of SEER (16; 17). The majority of these models are trained on the images (but not the labels) of ImageNet-1K or in the case of the SEER models, on 1 billion uncurated, unlabeled images gathered from a number of online sources. We include these models to assess the predictive power of features learned in the absence of explicit category supervision (or the implicit semantics thereof).
- The Taskonomy models consist of a core encoder-decoder architecture trained on 24 different common computer vision tasks, ranging from autoencoding to edge detection. These models are engineered in such a way that only the architecture of the decoder varies across task, allowing us to assess (after detaching the decoder) what effect different kinds of training has on predictive power, holding model architecture and training dataset constant.
- The CLIP models are models that consist of two encoders – one that processes images, and another that processes text associated with the images. These encoders learn simultaneously through a contrastive loss on their latent representations. This contrastive loss effectively coerces the embeddings of the text encoder and the embeddings of the vision encoder to the same vector. The features of the CLIP models are thus shaped by an abstracted, multimodal learning signal that constitutes a considerable shift away from pure perception, and a strong empirical foil to the purely perceptual models delineated above. The SLIP models (which directly compare multimodal supervision with unimodal self-supervision, as well as the combination of the two) provide an overall less performant version of the CLIP models, but with tighter empirical control on the images used for training and the training hyperparameters.

4 Explaining 'Explainable Variance Explained' as a Measure of Predictive Accuracy

The term "explainable variance explained" is a shortened version of the "Fraction of Explainable Variance Explained" (FEVE) metric proposed by Cadena et al. (18) for use in the prediction of brain activity by deep neural network models. This kind of metric (sometimes called a 'noise-normalized' score) is a now common metric in computational neuroscience (19) and is designed to give a more intuitive estimate of how accurate a model's predictions are given how accurate the model's predictions could be – an upper bound determined by the noise in the data the model is predicting (see (20; 21; 22) for examples in the neuroscience literature).

Noise-normalized accuracies can in theory be predicted over any scoring metric, but in practice are often calculated with the Pearson correlation between model-predicted and actual values (in our case, $r_{(y,\hat{y})}$) as the numerator and some form of correlation-based noise ceiling (in our case r_{split}) as the denominator. Here, we have opted to use the square of the correlation-based metric (r^2) to provide a measure of 'explained variance' (23). We have chosen r^2 as our measure of explained variance here both because it builds directly on our raw predictive accuracies (measured in units of $r_{Pearson}$) and also because (in the context of cross-validation) r^2 will often converge directly with R^2 (unless predictors are not centered, or there is systematic bias across the cross-validation folds (24) not otherwise accounted for by the intercept]).

As case in point, see Table 2: Here, we reproduce the model 'scores' (predictive accuracy) from Results Section A in units of r, r^2, r^2_{EVE} and the commonly used explained variance score (EV Score) from Scikit-Learn (effectively, an R^2 metric that does not adjust for bias in the intercept.) As the EV Score in this table makes clear, we indeed do not observe any systematic bias across our cross-validated predictions not otherwise accounted for by the intercept.

Table 2: Model 'Scores' (Accuracy) from Results Section A in Different Units

Model	Dataset	Rating	r (Ceiling)	r	r^2	EV Score	r^2_{EVE}
Mean	Oasis	Arousal	0.963	0.688	0.473	0.468	0.510
Mean	Oasis	Valence	0.992	0.663	0.440	0.433	0.447
Mean	Oasis	Beauty	0.989	0.746	0.556	0.552	0.569
Mean	Vessel	Beauty	0.862	0.671	0.452	0.449	0.606
Best	Oasis	Arousal	0.963	0.819	0.671	0.670	0.723
Best	Oasis	Valence	0.992	0.829	0.687	0.686	0.698
Best	Oasis	Beauty	0.989	0.874	0.764	0.764	0.781
Best	Vessel	Beauty	0.862	0.793	0.629	0.629	0.845

5 Taskonomy Model Results: Details + Discussion

In Figure 1, we show the full set of rankings for affect predictions across the Taskonomy models.

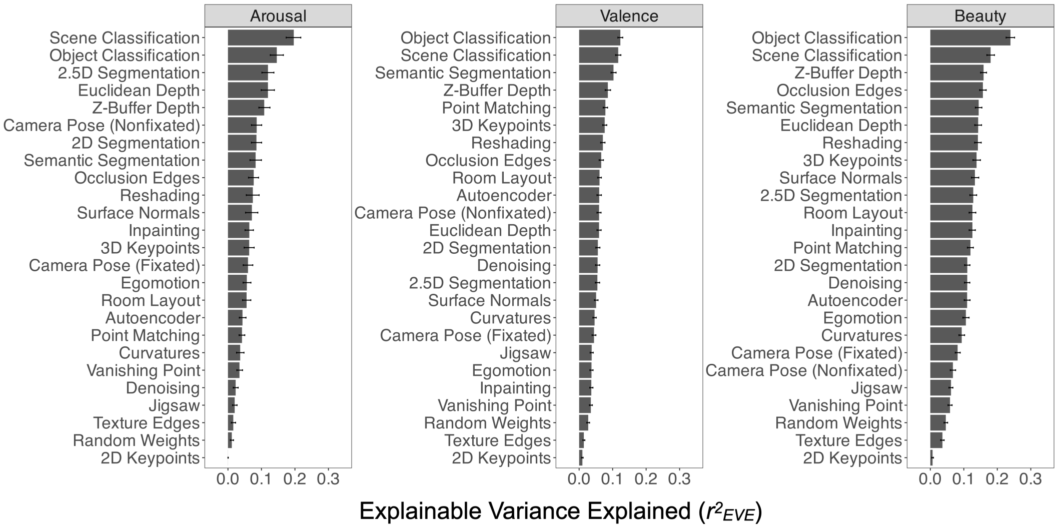

Figure 1: Results from the decoding of group-average affect ratings across the Taskonomy models (25), a set of 24 models that share the same architecture and visual diet, but are optimized on different training targets. The x-axis is in units of explainable variance explained. Error bars are the 95% bootstrapped confidence intervals from the resampling of the human respondent pool. Models trained on object and scene classification are the dominant models in this set, and rank 1st or 2nd in 1000 / 1000 bootstrap samples ($p < 0.001$). Notice, though, that the overall scores for these models (trained on a custom dataset consisting of 4.5 million indoor scenes) lags far behind the average of ImageNet-trained models, which (depending on affect) ranges from r^2_{EVE} = 0.511 - 0.606, underscoring the necessity of learning features tuned to a wider diversity of images for accurate prediction.

An important caveat when using the Taskonomy models in downstream comparisons to brain and behavioral data is their somewhat limited experience in terms of training over natural images. All the Taskonomy models are trained on the same 4.5 million indoor scenes, and while the raw quantity of images in this training set exceeds those of other datasets like ImageNet (with 1.2 million images), the *diversity* of these images is far more limited. This limited range of experience means first and foremost that we should be cautious when using these domain-specific models to make domain-general inferences about the affordances of computational task writ large. But it also underscores again the crucial point that the ability of any model to predict downstream targets like human affect is deeply contingent on the kinds of representations a model learns as a function of its sensory ecology and experience. (See (22) for a discussion of this point with respect to predictions of brain activity.)]

6 The Relationship Between Category Semantics and Affect Prediction

In both affective science and empirical aesthetics alike, there has long been considerable interest in understanding the relationship between recognition and feeling. Studies in this domain have inquired both whether such a relationship exists in the first place, and if it does, whether that relationship is casual, bidirectional, incidental, or epiphenomenal. Some examples from affective science include the century-old 'bear in a forest' thought experiments posed by William James (26), the observation of fear responses in pre-linguistic infants to dangerous (e.g. 'snake-like') objects (27), and the analysis of dissociations between object and emotion processing in individuals with aphasias or other forms of neuropsychological deficits (28; 29). In empirical aesthetics, there has recently been a renewed push to more directly link the experience of beauty (or 'sensory reward') to category learning (30; 31; 32; 33). One of the primary conceptual difficulties at play in this work is the same difficulty that challenges the disentanglement of perception and affect with brain or behavioral analyses alone – namely, that the 'meaning' (semantics) of a nameable thing is composed both of 'what that thing is' (its category) and 'what that thing makes us feel' (its associated affect).

While it may eventually possible to use affectless machine vision models to pull these two kinds of meaning apart, our analysis of category-supervised versus self-supervised models shows that doing so may require re-conceptualizing what exactly a category is at the level of neural population codes. If, for example, a category may be understood as a distribution of learned features that can be translated to a single label by way of single, linear transforms (i.e. read-out), the difference between category-supervised and self-supervised models becomes less about the presence or absence of category information, and more about how that information got there (i.e. how it was learned): by 'top-down' or 'bottom-up' processes. This is, in fact, the primary lens by which the interpret the difference between self- and category-supervised models in our own work. The category-learning in self-supervised models is 'bottom-up'. The category-learning in category-supervised models is 'top-down'. And because self-supervised models predict affect as accurately category-supervised models, we can again say with some confidence that 'bottom-up' perceptual learning is a viable, candidate driver of downstream affective processes.

Nevertheless, one lingering concern may be that the affect predictions of perceptual models (self- or category-supervised) in our analyses may effectively reduce to simple conceptual mappings like "(dog, positive)", "(cat, negative)". The logic of 'top-down' versus 'bottom-up' learning applies equally to this edge case, but it does leave open the possibility of a confound in our image set that might artificially inflate our sense of the models' overall accuracy in predicting affect. One way we can at least partially address these concerns is by using the image labels (provided with OASIS) as predictors in the same regression pipeline we have so far applied to the deep neural network features. The logic here is that if the variance in affect ratings is accounted for by the OASIS image labels, and if the models have learned features that adequately approximate these labels, then predictions by label alone may suffice to approximate the model predictions. We find this is not the case: Converted to binary features using one-hot dummy coding, the OASIS image labels (e.g. "beach", "flowers", "soldiers", or "zebra") yield predictive accuracies of $r_{(y,\hat{y})}$ = 0.245 for arousal, 0.247 for valence, and 0.236 for beauty. The *least-predictive* supervised model (ResNet50-Jigsaw or RotNet) yields 0.532 for arousal, 0.459 for 0.598. In short, affect prediction by category alone is nontrivial, but less than half as accurate as self-supervised model features. Denser category labels might close this gap, but would also bring us closer to the regime of the vision-language tradeoff we elsewhere designate as beyond the scope of this analysis.

7 On the Relationship of Inter-Rater Agreement to Affect Prediction

In our main analysis, we suggest that the differences in the ability of the purely perceptual models to predict affect across image category are most likely to be a function of difference in inter-rater agreement: In the Vessel dataset, for example, raters tend to agree most substantially in their ratings of landscapes, and disagree most substantially in their ratings of art. One alternative explanation is that this difference is more directly attributable to differences in the aggregate statistical properties of the images themselves – especially in terms of their homogeneity. In this formulation, ratings for landscapes are more easily predicted because their features are more homogeneous; ratings for art are less easily predicted because their features are less homogeneous. Previous work has shown that the features of DNN models may indeed be sensitive to these differences (34).

Assuming for the sake of the discourse that this difference between image categories is true for both the datasets in our analysis (OASIS and the Vessel dataset), we believe inter-rater agreement still remains the more likely explanation of the difference for the following reasons: In the original Vessel et al. (35) data, the authors found that test-retest scores were not different between landscape and artwork sets, which suggests that neither set was necessarily more "confusable" than the other, and therefore not more "homogeneous" (or heterogeneous) in whatever internal representational space that raters were using to make their judgments. One could also argue, especially in the case of modeling, that a more heterogeneous stimulus set, with more discernible features, would lead to more robust predictions, as opposed to a stimulus set with lower variation on fewer dimensions (which would entail higher average similarity across the set). And indeed, such a phenomenon has already been demonstrated in the empirical aesthetics literature, with findings that show a *negative* relationship between the similarity of abstract artwork and inter-rater agreement (36; 37; 38) (the more similar the set, the lower the agreement).

Another advantage of the inter-rater agreement explanation for the difference of perceptual affect prediction across image category is how it allows us to interface with the idea of judgments that are based on 'more' or 'less' perceptual criteria. In this formulation, art may be less predictable by purely perceptual models than landscapes because art more readily evokes higher-order 'conceptual' information that purely perceptual models cannot learn. Art that evokes ideas of 'justice', 'love', or 'spirituality' – ideas not so easily associated with common, grounded imagery – are in many ways evoking properties that may not even be learnable through feedforward visual learning at all. Purely 'perceptual' models are, in this sense, models of commonly held visual priors. The more certain kinds of images depart from these priors, the less likely human raters will be to agree about what they mean and how they feel in response; the less likely these raters are to agree on the basis of perceptual priors, the more likely it is that 'perceptual' models will fail to predict their ratings.

8 On the Relationship of Taste-Typicality to Affect Prediction

The positive correlations between each respondent's 'mean-minus-one' (r_{MM1}) correlation and predictability, while significant, are exploratory findings that merit further scrutiny with future experimentation. Key to assessing whether this result is meaningful will be a better estimate of each respondents' individual consistency across consecutive ratings of the same image, or multiple estimates of that respondent's responses by means of different (multimodal) prompts (i.e. keypress ratings that are used in certain experiments (35) to provide an alternative to self-report). Without this measure, we cannot discount the possibility that respondents whose response profiles deviate from the group-average did not complete the task as instructed.

With this in mind, it may still be worthwhile to consider the implications of better decoding performance for respondents who are more taste-typical – what we might provisionally call 'automated hipster detection'. Interpreting this result is most straightforward perhaps in terms of beauty – the affect rating in which the effect is strongest. Respondents with tastes more grounded in shared perceptual priors are likely to be those respondents better predicted by the purely perceptual models in our survey; respondents whose tastes deviate due to cultural or cognitive idiosyncrasies are difficult (if not by definition impossible) to predict without models further shaped by these factors. There is strong evidence to suggest that 'taste-typicality' is a foundational axis of aesthetic experience that bridges sensory modalities (39), and it may well manifest in the ratings we have assessed here.

9 Additional Details on Gains from Language-Alignment

A figure summarizing the controlled assessment of gains in affect prediction attributable specifically to the task of language alignment (using the SLIP models) (13) may be found in Figure 2 below:

Domain-Specific Gains In Figure 3 below, we show details of the gains made by language-aligned vision models across affect and image category.

OpenAI's CLIP Compared to Other Models Trained on Larger Numbers of Images While the SLIP models are by far the more controlled test of the influence of language, holding number of training images constant, OpenAI's CLIP still outperforms these by a large margin. Could this gap be attributable to larger quantities of training images alone?

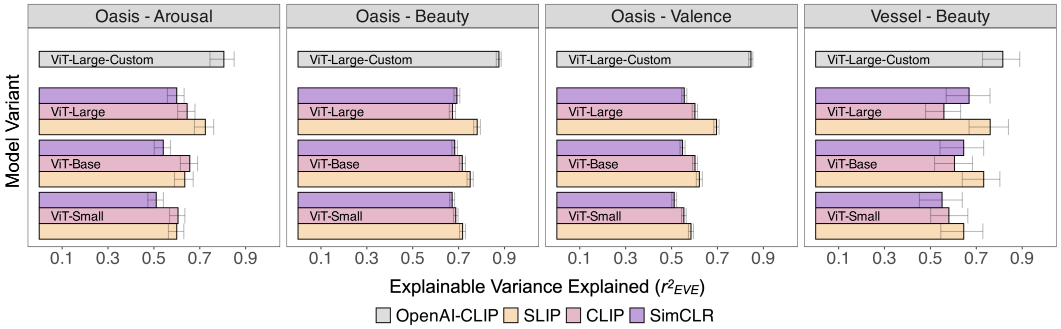

Figure 2: Extending the paradigm of decoding beyond purely perceptual models to models that learn from vision and language simultaneously: This plot shows the performance of OpenAI's most performant CLIP model (in gray), and a series of 9 models from FaceBook's SLIP repository, which contrasts pure self-supervision (SimCLR-style) with pure language contrastive learning (CLIP-style) and a combination of the two (SLIP). The SLIP models allow us to better disentangle the effects of various modeling hyperparameters (model architecture, number of training images) from the theoretically relevant contrast of learning with or without language. Here, we see that (at least when training on the 15 million images of the YFCC100M image set) the advantage of language training mostly manifests in conjunction with the augmentations of contrastive self-supervision, though does provide a discernible advantage over self-supervision (perception) alone in ratings of beauty and valence in the Oasis dataset.

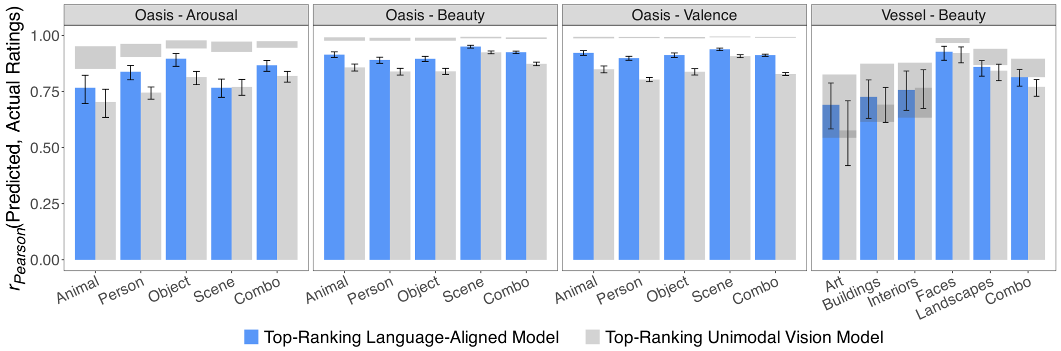

Figure 3: Gain in predictivity as a function of natural language supervision across all distinct combinations of affect and image category. These gains are most substantial in predicting ratings of Beauty for Art in the Vessel dataset. Error bars are again the 95% bootstrapped confidence intervals from 1000 resamples of the human respondent pool. The shaded crossbars are the 95% confidence interval on the Spearman-Brown splithalf-reliability of the target affect ratings.

To answer this, we surveyed an additional subset of models with a greater amount of training samples than are available in ImageNet. Google's Big Transfer (BiT) models are a series of standard ResNets pretrained on Google's proprietary JFT-300M (300 million image) dataset. The most performant BiT model we test (ResNet101-V2-101x3, further finetuned on ImageNet21K) achieves a bootstrapped average explained variance score of 0.66 [0.631, 0.686] (slightly less than even the Swin transformer trained only on standard ImageNet). FaceBook's semi-weakly-supervised ResNet50, trained on approximately 900 million images (2.5x the number of images in the CLIP training set) achieves an average of 0.652 [0.629, 0.671] (substantially more than ImageNet-trained ResNet50's average of 0.473 [0.449, 0.498], but substantially less then CLIP-trained ResNet50's average of 0.781 [0.756, 0.802]). Most compelling, perhaps, are the SEER models: state-of-the-art self-supervised models trained on a billion images. The most performant of these (a RegNet-128Gf architecture) obtains an average of 0.781 [0.756, 0.802].

These models should not be considered true empirical contrasts to CLIP – as they differ in more than the explanatory variables of interest (that is, the number of images in the training set, and the optimization function). Unlike the Taskonomy models and the majority of the ImageNet-trained

models, the testing of these models constitutes a somewhat more uncontrolled set of naturalistic experiments, made possible by the availability of these pretrained models for public use. However, they do give an initial indication that the number of images in the training set is not the *sole* factor that drives CLIP's augmented predictivity. Instead (and in concert with the SLIP results), these results suggest CLIP's advantage may be an *interaction* of training set and the language-alignment task.

References

[1] Naila Murray, Luca Marchesotti, and Florent Perronnin. Ava: A large-scale database for aesthetic visual analysis. In *2012 IEEE conference on computer vision and pattern recognition*, pages 2408–2415. IEEE, 2012.

[2] Benedek Kurdi, Shayn Lozano, and Mahzarin R Banaji. Introducing the open affective standardized image set (oasis). *Behavior research methods*, 49(2):457–470, 2017.

[3] Aenne A Brielmann and Denis G Pelli. Beauty requires thought. *Current Biology*, 27(10): 1506–1513, 2017.

[4] Alex Krizhevsky, Ilya Sutskever, and Geoffrey E Hinton. ImageNet classification with deep convolutional neural networks. In *Advances in Neural Information Processing Systems*, pages 1097–1105, 2012.

[5] Karen Simonyan and Andrew Zisserman. Very deep convolutional networks for large-scale image recognition. *arXiv preprint arXiv:1409.1556*, 2014.

[6] Kaiming He, Xiangyu Zhang, Shaoqing Ren, and Jian Sun. Deep residual learning for image recognition. In *Conference on Computer Vision and Pattern Recognition*, pages 770–778, 2016.

[7] Alexey Dosovitskiy, Lucas Beyer, Alexander Kolesnikov, Dirk Weissenborn, Xiaohua Zhai, Thomas Unterthiner, Mostafa Dehghani, Matthias Minderer, Georg Heigold, Sylvain Gelly, Jakob Uszkoreit, and Neil Houlsby. An image is worth 16x16 words: Transformers for image recognition at scale, 2020.

[8] Ze Liu, Yutong Lin, Yue Cao, Han Hu, Yixuan Wei, Zheng Zhang, Stephen Lin, and Baining Guo. Swin transformer: Hierarchical vision transformer using shifted windows, 2021.

[9] Zhuang Liu, Hanzi Mao, Chao-Yuan Wu, Christoph Feichtenhofer, Trevor Darrell, and Saining Xie. A convnet for the 2020s. In *Proceedings of the IEEE/CVF Conference on Computer Vision and Pattern Recognition*, pages 11976–11986, 2022.

[10] Ting Chen, Simon Kornblith, Mohammad Norouzi, and Geoffrey Hinton. A simple framework for contrastive learning of visual representations. In *International Conference on Machine Learning*, pages 1597–1607. PMLR, 2020. arXiv preprint arXiv:2002.05709.

[11] Mathilde Caron, Hugo Touvron, Ishan Misra, Hervé Jégou, Julien Mairal, Piotr Bojanowski, and Armand Joulin. Emerging properties in self-supervised vision transformers. In *Proceedings of the International Conference on Computer Vision (ICCV)*, 2021.

[12] Alec Radford, Jong Wook Kim, Chris Hallacy, Aditya Ramesh, Gabriel Goh, Sandhini Agarwal, Girish Sastry, Amanda Askell, Pamela Mishkin, Jack Clark, et al. Learning transferable visual models from natural language supervision. In *International Conference on Machine Learning*, pages 8748–8763. PMLR, 2021.

[13] Norman Mu, Alexander Kirillov, David Wagner, and Saining Xie. Slip: Self-supervision meets language-image pre-training. *arXiv preprint arXiv:2112.12750*, 2021.

[14] Priya Goyal, Quentin Duval, Jeremy Reizenstein, Matthew Leavitt, Min Xu, Benjamin Lefaudeux, Mannat Singh, Vinicius Reis, Mathilde Caron, Piotr Bojanowski, Armand Joulin, and Ishan Misra. Vissl. `https://github.com/facebookresearch/vissl`, 2021.

[15] Aenne A Brielmann and Denis G Pelli. Intense beauty requires intense pleasure. *Frontiers in psychology*, 10:2420, 2019.

[16] Priya Goyal, Mathilde Caron, Benjamin Lefaudeux, Min Xu, Pengchao Wang, Vivek Pai, Mannat Singh, Vitaliy Liptchinsky, Ishan Misra, Armand Joulin, et al. Self-supervised pretraining of visual features in the wild. *arXiv preprint arXiv:2103.01988*, 2021.

[17] Priya Goyal, Quentin Duval, Isaac Seessel, Mathilde Caron, Mannat Singh, Ishan Misra, Levent Sagun, Armand Joulin, and Piotr Bojanowski. Vision models are more robust and fair when pretrained on uncurated images without supervision. *arXiv preprint arXiv:2202.08360*, 2022.

[18] Santiago A Cadena, George H Denfield, Edgar Y Walker, Leon A Gatys, Andreas S Tolias, Matthias Bethge, and Alexander S Ecker. Deep convolutional models improve predictions of macaque v1 responses to natural images. *PLoS computational biology*, 15(4):e1006897, 2019.

[19] Dean A Pospisil and Wyeth Bair. The unbiased estimation of the fraction of variance explained by a model. *PLoS computational biology*, 17(8):e1009212, 2021.

[20] Umut Güçlü and Marcel AJ van Gerven. Deep neural networks reveal a gradient in the complexity of neural representations across the ventral stream. *Journal of Neuroscience*, 35(27): 10005–10014, 2015. doi: 10.1523/JNEUROSCI.5023-14.2015. Publisher: Soc Neuroscience.

[21] Chengxu Zhuang, Siming Yan, Aran Nayebi, Martin Schrimpf, Michael C Frank, James J DiCarlo, and Daniel LK Yamins. Unsupervised neural network models of the ventral visual stream. *Proceedings of the National Academy of Sciences*, 118(3), 2021.

[22] Colin Conwell, Jacob S Prince, Kendrick N Kay, George A Alvarez, and Talia Konkle. What can 1.8 billion regressions tell us about the pressures shaping high-level visual representation in brains and machines? *BioRxiv*, pages 2022–03, 2023.

[23] Michael H Kutner, Christopher J Nachtsheim, John Neter, and William Li. *Applied linear statistical models*. McGraw-hill, 2005.

[24] Gareth James, Daniela Witten, Trevor Hastie, Robert Tibshirani, et al. *An introduction to statistical learning*, volume 112. Springer, 2013.

[25] Amir R Zamir, Alexander Sax, William Shen, Leonidas J Guibas, Jitendra Malik, and Silvio Savarese. Taskonomy: Disentangling task transfer learning. In *Proceedings of the IEEE Conference on Computer Vision and Pattern Recognition*, pages 3712–3722, 2018.

[26] William James, Frederick Burkhardt, Fredson Bowers, and Ignas K Skrupskelis. *The principles of psychology*, volume 1. Macmillan London, 1890.

[27] Judy S DeLoache and Vanessa LoBue. The narrow fellow in the grass: Human infants associate snakes and fear. *Developmental science*, 12(1):201–207, 2009.

[28] Susan Sullivan and Ted Ruffman. Emotion recognition deficits in the elderly. *International Journal of Neuroscience*, 114(3):403–432, 2004.

[29] Maxime Bertoux, Harmony Duclos, Marie Caillaud, Shailendra Segobin, Catherine Merck, Vincent de La Sayette, Serge Belliard, Béatrice Desgranges, Francis Eustache, and Mickaël Laisney. When affect overlaps with concept: Emotion recognition in semantic variant of primary progressive aphasia. *Brain*, 143(12):3850–3864, 2020.

[30] Anjan Chatterjee and Eileen Cardilo. *Brain, beauty, and art: Essays bringing neuroaesthetics into focus*. Oxford University Press, 2021.

[31] Aenne A Brielmann and Peter Dayan. A computational model of aesthetic value. *Psychological review*, 129(6):1319, 2022.

[32] Delaram Farzanfar and Dirk B Walther. Changing what you like: Modifying contour properties shifts aesthetic valuations of scenes. *Psychological Science*, 34(10):1101–1120, 2023.

[33] Gaeun Son, Dirk B Walther, and Michael L Mack. Brief category learning distorts perceptual space for complex scenes. *Psychonomic Bulletin & Review*, pages 1–15, 2024.

[34] Anselm Brachmann, Erhardt Barth, and Christoph Redies. Using CNN features to better understand what makes visual artworks special. *Frontiers in Psychology*, 8:830, 2017. doi: 10.3389/fpsyg.2017.00830. Publisher: Frontiers.

[35] Edward A Vessel, Natalia Maurer, Alexander H Denker, and G Gabrielle Starr. Stronger shared taste for natural aesthetic domains than for artifacts of human culture. *Cognition*, 179:121–131, 2018.

[36] Edward A. Vessel. Beauty and the beholder: Highly individual taste for abstract, but not real-world images. *Journal of Vision*, 10(2):1–14, 2010. ISSN 15347362. doi: 10.1167/10.2.18. URL http://jov.arvojournals.org/Article.aspx?doi=10.1167/10.2.18.

[37] Astrid Schepman, Paul Rodway, Sarah J Pullen, and Julie Kirkham. Shared liking and association valence for representational art but not abstract art. *Journal of Vision*, 15:1–10, 2015. ISSN 1534-7362. doi: 10.1167/15.5.11.doi.

[38] Astrid Schepman, Paul Rodway, and Sarah J Pullen. Greater cross-viewer similarity of semantic associations for representational than for abstract artworks. *Journal of Vision*, 15:1–6, 2015. doi: 10.1167/15.14.12.doi.

[39] Yi-Chia Chen, Andrew Chang, Monica D Rosenberg, Derek Feng, Brian J Scholl, and Laurel J Trainor. "taste typicality" is a foundational and multi-modal dimension of ordinary aesthetic experience. *Current Biology*, 2022.
